# Construction of *N*-Ferrocene Substituted Benzodihydrooxazoles via a Catalyst-Free Aza-Michael Addition/C(sp^3^)-O Bond Formation Tandem Reaction

**DOI:** 10.3390/molecules28145615

**Published:** 2023-07-24

**Authors:** Mingliang Zhang, Pin Zhao, Qilv Liu, Xinlei Liu, Jingya Hu, Dongqing Wu, Lantao Liu

**Affiliations:** 1 Henan Engineering Research Center of Green Synthesis for Pharmaceuticals, School of Chemistry and Chemical Engineering, Shangqiu Normal University, Shangqiu 476000, China; moonlightzhang@163.com (M.Z.);; 2 College of Chemistry, Zhengzhou University, Zhengzhou 450052, China; 13623702703@163.com

**Keywords:** amino ferrocenes, benzodihydrooxazole, C(sp^3^)-O bond formation, aza-Michael addition, catalyst-free

## Abstract

A catalyst-free aza-Michael addition/C(sp^3^)-O bond formation tandem reaction of substituted amino ferrocenes with quinone esters was developed, which provided a green and efficient strategy for the construction of a C(sp^3^)-O bond from C(sp^3^)-H, and a series of *N*-ferrocene-substituted benzodihydrooxazoles were smoothly produced in moderate to excellent yields (up to >99% yield). The mechanism experiments showed that quinone esters performed as both substrate and oxidant. The salient features of this transformation include good functional group tolerance, broad substrate scope and mild conditions.

## 1. Introduction

**Scheme 1:**
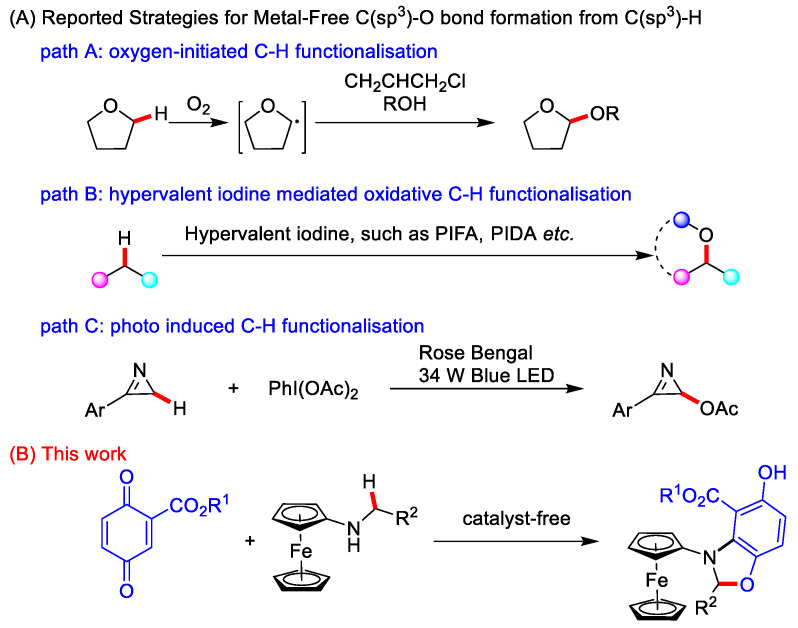
(**A**) Reported metal-free strategies for the construction of the C(sp^3^)-O bond from C(sp^3^)-H. (**B**) Synthetic design in this work.

Intramolecular C(sp^3^)-O bond formation, which is widely used in the synthesis of natural products, drugs and other functional molecules, has emerged as one of the most powerful strategies to access oxygen-containing heterocycles [1,2,3]. In this respect, much attention has been paid to exploring refined strategies for the construction of the C(sp^3^)-O bond, among which transition metal-catalyzed oxidation of C(sp^3^)-H is the main strategy and has been well developed [4,5,6,7]. However, this method suffers from high costs, harsh reaction conditions and transition metal residues. In view of the growing problems due to climate and environmental change, chemists intend to explore metal-free protocols for the construction of the C(sp^3^)-O bond from C(sp^3^)-H which are in line with green chemistry and sustainable development concepts. However, this area is underdeveloped due to the lower nucleophilicity of oxygen compared with nitrogen [8,9]. Until now only three approaches are capable of performing such transformations. The first protocol is aerobic-initiated C(sp^3^)-H bond oxidation (Scheme 1A, path A). In 2010, Troisi et al. reported the direct C(sp^3^)-H bond oxidation of heating tetrahydrofuran in the presence of air and allyl or benzyl chloride [10]. Another access is hypervalent iodine mediated C(sp^3^)-H bond oxidation (Scheme 1A, path B). Hypervalent iodine reagents have been widely used as alternatives to transition metals due to their high reactivity and environmentally friendly properties. The Fan group, Du and Zhao group, Dominguez group and Martin group realized the construction of the C(sp^3^)-O bond by direct oxidation of C(sp^3^)-H with phenyliodine diacetate (PIDA), phenyliodine bis(trifluoroacetate) (PIFA) or iodobenzene peroxide as oxidation systems [11,12,13,14,15,16,17]. The third method for the metal free construction of the C(sp^3^)-O bond is photo-induced C(sp^3^)-H functionalization (Scheme 1A, path C). In 2019, Majee et al. developed visible-light-promoted regioselective C(sp^3^)-H acyloxylation of aryl-2H-azirine with phenyliodine diacetate using Rose Bengal as organophotoredox catalyst [18]. Later in 2020, Ohmiya and coworkers reported a visible-light-mediated decarboxylative coupling between aliphatic alcohol and alkyl carboxylic acid-derived redox active esters [19]. These excellent works provide a variety of effective strategies for the construction of the C(sp^3^)-O bond from direct oxidation of the C(sp^3^)-H bond, but additional oxidants, catalysts or free radical initiators are required. Therefore, exploiting a simple and more efficient protocol for the construction of the C(sp^3^)-O bond through direct oxidation of the C(sp^3^)-H bond without catalyst participation is of great significance.

Benzodihydrooxazoles are an important class of nitrogen and oxygen-containing heterocycles which are prevalent in natural products, bioactive molecules and many other functional molecules [20,21,22,23,24,25]. Considerable efforts have been devoted to the synthesis of this unique skeleton, strategies such as cyclization of 2-aminophenol, transition-metal-catalyzed intramolecular C-H amination reactions and coupling reactions of benzoxazoles have been discussed [22,26,27,28,29,30,31,32,33,34,35,36]. However, the drawbacks of these methods are the requirement of strong acids or bases, participation of transition metal catalysts, lengthy steps and high reaction temperatures. Considering the wide application of benzodihydrooxazoles, exploiting strategies with mild reaction conditions and simple and efficient reaction systems are still in high demand.

Functional molecules containing ferrocene scaffolds are widely applied in medicinal chemistry, materials science and asymmetric synthesis [37,38,39,40,41,42,43,44]. Ferrocene plays an important role and is recognized as the core scaffold of organocatalysts and chiral ligands, especially in the field of asymmetric synthesis. Due to its unique sandwich structure and electronic properties, the introduction of ferrocene into functional molecules is an attractive approach for improving the properties of these molecules [45,46,47,48,49,50]. Combination of two functional molecules is also a common strategy in the construction of novel dominant skeletons. Therefore, a molecule containing both a ferrocene and benzodihydrooxazole skeleton may be a new type of privileged functional molecule. 

Tandem reactions, in which multiple transformations are combined in a single procedural step, have been widely employed for the construction of complex molecules [51,52,53,54,55,56,57,58]. In view of the inherent advantages of tandem reactions, we decided to apply this strategy to the synthesis of compounds bearing both ferrocene and benzodihydrooxazole moieties. As part of our ongoing interest in the construction ferrocene-based compounds and heterocycles [59,60,61,62,63], we present herein a catalyst-free tandem reaction of quinone esters with substituted amino ferrocene derivatives through an aza-Michael addition/C(sp^3^)-O bond formation process without additional oxidants; a series of *N*-ferrocene-substituted benzodihydrooxazoles were produced in moderate to excellent yields.

## 2. Results and Discussion

### 2.1. Optimization Studies

Initially, we investigated the reaction of quinone ester **1a** and *N*-benzyl amino ferrocene **2a** under the catalysis of DABCO in DCM at 35 °C for 17 h. Encouragingly, the desired product was obtained in a 67% yield (Table 1, entry 1). When the reaction was performed with DMAP as a catalyst, the expected product was isolated only in a 22% yield (Table 1, entry 2). Brønsted acids, such as diphenyl phosphate, TsOH and benzoic acid, were also employed as catalysts in pursuit of high yields, and diphenyl phosphate gave a better result in an 85% yield (Table 1, entries 3–5). Surprisingly, the product was produced with a quantitative yield in the absence of a catalyst (Table 1, entry 6). The effects of different solvents was also investigated. When the reaction was conducted in acetonitrile, ethyl acetate, tetrahydrofuran and toluene, relatively lower yields were obtained (Table 1, entries 7–10). Reaction temperature and substrate ration were also investigated, lower or higher temperature and substrate ration gave inferior results (see Supplementary Materials). In consequence, we identified the following optimal conditions: **1a** (0.10 mmol) and **2a** (0.05 mmol) in 0.5 mL of DCM were stirred at 35 °C for 17 h.

### 2.2. Substrate Scope Studies

**Scheme 2:**
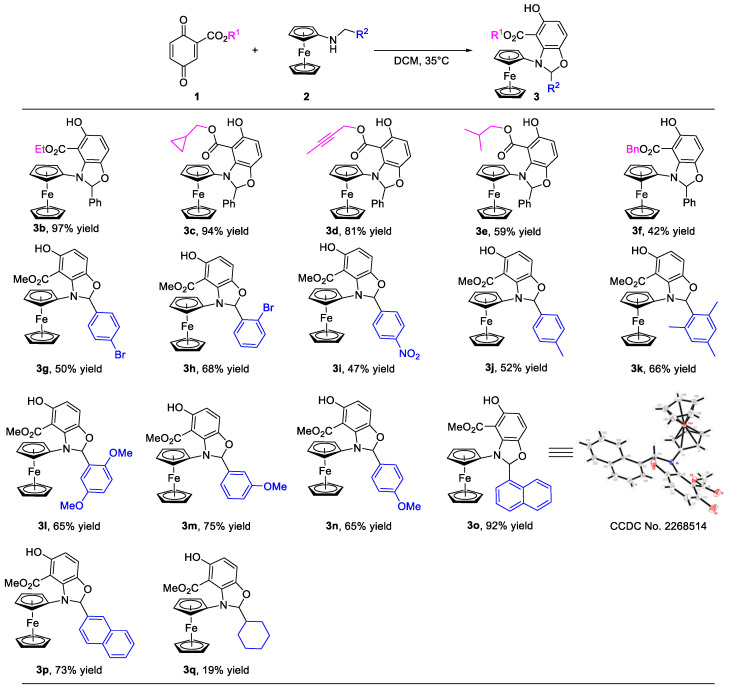
Substrate scope of the reaction. Reaction conditions: **1** (0.10 mmol) and **2** (0.05 mmol) were stirred in DCM (0.5 mL) at 35 °C for 17 h. The yields refer to the isolated yields.

**Scheme 3:**
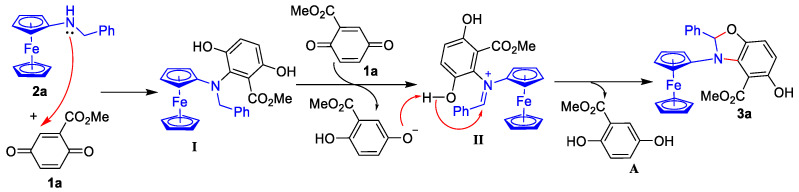
Proposed mechanism for the catalyst-free aza-Michael addition/C(sp^3^)-O bond formation tandem reaction.

The reaction scope of this reaction was evaluated with respect to both the quinone esters **1** and the substituted amino ferrocenes **2** with optimized reaction conditions. First, the substituent R^1^ of quinone esters **1** was examined. The desired products were obtained in yields of 81–97% when R^1^ is an ethyl, cyclopropylmethyl or but-2-yn-1-yl group (Scheme 2, **3b**–**3d**). While isobutyl and benzyl groups gave obviously lower yields of 59% and 42%, respectively (Scheme 2, **3e** and **3f**). Then the substituent R^2^ of amino ferrocenes **2** was also evaluated. When R^2^ are substituted phenyl groups, the desired products were obtained in 47–75% yields, and the electronic nature or position of the substituents on the phenyl ring obviously affected the reaction. Both 4-Nitrophenyl and 4-bromophenyl groups gave the expected products at yields of 50% and 47%, respectively (Scheme 2, **3g**, **3i**). The yield increased to 68% when Br was installed in the *ortho*-position of the phenyl ring (Scheme 2, **3h**). The electronic nature, position and the number of electron-donating groups on the phenyl ring also have noticeable influence on the reaction. The corresponding products were produced in a 52% yield when R^2^ is a *p*-tolyl group (Scheme 2, **3j**); dramatically increased yields (65–75%) were produced when a methoxy group or multiple electro-donating substituents were installed on the phenyl ring (Scheme 2, **3k**–**3n**). A naphthyl group is also well tolerated for this transformation. While the position of the substituent significantly affected the yields, a 1-naphthyl group gave the expected product in a 92% yield while only a 73% yield was obtained when R^2^ is a 2-naphthyl group (Scheme 2, **3o** and **3p**). An alkyl substituent was also tolerated for this reaction, despite relatively low yields being obtained (Scheme 2, **3q**). The structure of **3o** was confirmed by X-ray crystallographic diffraction analysis (CCDC 2268514) and those of other products were assigned by analogy.

### 2.3. Proposed Mechanism for the Catalyst-Free Aza-Michael Addition/C(sp^3^)-O Bond Formation Tandem Reaction

**Figure 1:**
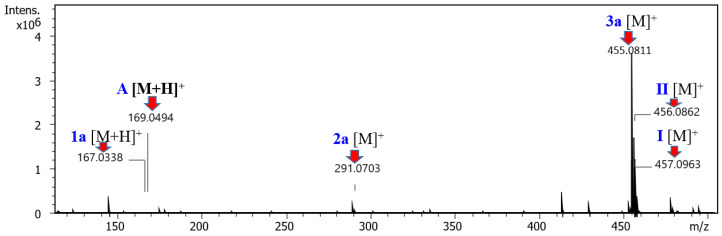
HRMS spectra of the crude reaction mixture between **1a** and **2a**.

Based on the experimental results and previous reports [64], a possible reaction process for this transformation was proposed as demonstrated in Scheme 3. Initiated by the aza-Michael addition of quinone ester **1a** and *N*-benzyl amino ferrocene **2a**, the formed intermediate **I** was oxidized to intermediate **II** by quinone ester **1a**. Finally, the desired product **3a** was obtained from intermediate **II** through intramolecular C-O bond formation. To shed light on the mechanism of this reaction, HRMS analysis of the crude reaction mixture of quinone ester **1a** and *N*-benzyl amino ferrocene **2a** was performed (Figure 1). All the signal peaks of intermediate **I**, **II**, and methyl 2,5-dihydroxybenzoate (**A**) were detected.

## 3. Materials and Methods

### 3.1. General Information

Reagents were purchased from commercial sources and were used as received unless mentioned otherwise. Reactions were monitored by thin layer chromatography (TLC). ^1^H NMR (400 MHz) and ^13^C NMR (101 MHz) spectra were recorded on a Bruker 400 spectrometer. Chemical shifts reported in parts per million (ppm) referred to tetramethylsilane (0.00 ppm) or residues of CDCl_3_ (7.26 ppm). Data are reported as follows: chemical shift, multiplicity (s = singlet, d = doublet, t = triplet, q = quartet, m = multiplet), coupling constants (Hz) and integration. Mass spectra (HRMS) were collected on a quadrupole time-of-flight mass spectrometer (Bruker Impact II, Bremen, Germany). Melting points were obtained on a SGW X-4 melting point apparatus. All solvents used were distilled with standard techniques. Single crystal was recorded on a Gemini E diffractometer.

### 3.2. Method for Crystal Growth of ***3o***

A total of 5.0 mg of compound **3o** dissolved in 1 mL dichloromethane and 10 mL petroleum ether was added to a 20 mL sample vial, and brown yellow crystals were obtained after slow evaporation at 25 °C for several days.

### 3.3. General Experimental Procedure for the Catalyst-Free Aza-Michael Addition/C(sp^3^)-O Bond Formation Tandem Reaction for the Synthesis of Products ***3***

Quinone ester **1** (0.10 mmol, 2.0 equiv.) and amino ferrocene **2** (0.05 mmol, 1.0 equiv.) were dissolved in dichloromethane (0.5 mL) in a test tube. The mixture was stirred at 35 °C in an oil bath and monitored by thin-layer chromatography (TLC). Upon completion of the reaction, the mixture was charged onto a silica gel column directly, and the desired product was purified by flash chromatography with petroleum ether/ethyl acetate (*v*/*v* = 15:1) as an eluent.

## 4. Conclusions

In conclusion, a catalyst-free aza-Michael addition/C(sp^3^)-O bond formation tandem reaction of quinone esters with amino ferrocene derivatives was realized, which provided a green and efficient strategy for the construction of the C(sp^3^)-O bond from C(sp^3^)-H, and gave a series of *N*-ferrocene-substituted benzodihydrooxazoles in moderate to excellent yields. The salient features of this transformation include good functional group tolerance, broad substrate scope and mild conditions. The mechanism experiments showed that quinone esters **1** performed as both substrate and oxidant in the reaction.

## Figures and Tables

**Table 1:** Optimization of reaction conditions [a].

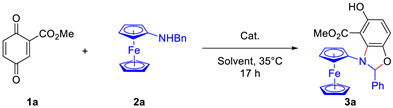			
Entry	Solvent	Cat.	Yield (%) [b]
1	DCM	DABCO	67
2	DCM	DMAP	22
3	DCM	(PhO)_2_PO_2_H	85
4	DCM	TsOH	45
5	DCM	PhCO_2_H	75
6	DCM	-	>99
7	CH_3_CN	-	50
8	EtOAc	-	67
9	THF	-	35
10	toluene	-	83

[a] Unless otherwise noted, the reaction was carried out with **1a** (0.10 mmol), **2a** (0.05 mmol), catalyst (0.01 mmol), solvent (0.5 mL) at 35 °C for 17 h. [b] Isolated yield. DABCO = 1,4-diazabicyclo [2.2.2]octane; DCM = dichloromethane; DMAP = *N*,*N*-4-dimethylaminopyridine.

## Data Availability

All the required data are reported in the manuscript and Supplementary Materials.

## Supplementary Materials

The following supporting information can be downloaded at: https://www.mdpi.com/article/10.3390/molecules28145615/s1, Characterization data for obtained products; copies of ^1^H and ^13^C NMR.

## Sample Availability

Samples of the compounds are available from the authors.
