# Supplementary material for: Construction of N-Ferrocene Substituted Benzodihydrooxazoles via a Catalyst-Free Aza-Michael Addition/C(sp3)-O Bond Formation Tandem Reaction
Source: Molecules. 2023 Jul 24;28(14):5615. doi: 10.3390/molecules28145615 (PMC10384804; doi:10.3390/molecules28145615)

Suporting Information for

Construction of *N*-ferrocene Substituted Benzodihydrooxazole via Catalyst-free Aza-Michael Addition/C(sp^3)-O Bond Formation Tandem Reaction

Zhang, Mingliang[†, a] Zhao, Pin[†, b] Liu, Qilv[a] Liu, Xinlei[a] Hu, Jingya[a] Wu, Dongqing*[,a] Liu, Lantao*[,a,b]

([a] School of Chemistry and Chemical Engineering, Henan Engineering Research Center of Green Synthesis for Pharmaceuticals, Shangqiu Normal University, Shangqiu, 476000)

([b] College of Chemistry, Zhengzhou University, Zhengzhou 450052)

*E-mail: liult05@iccas.ac.cn; iamwudongqing@163.om

Table of contents

1. General Information....2
2. Optimization of reaction conditions....2
3. General Procedure for Catalyst-free Aza-Michael Addition/C(sp^3)-O Bond Formation Tandem Reaction....2
3. Characterizations of Compounds **3**....3
5. NMR Spectra for compounds **3**....7

1. General Information

^{1}H NMR (400 MHz), ^{13}C NMR (101 MHz) spectra were recorded on a Bruker 400 spectrometer. Chemical shifts were reported in parts per million (ppm) referenced to tetramethylsilane (0.00 ppm) or residue of $CDCl_3$ (7.26 ppm). Mass spectra (HRMS) were collected on a quadrupole time-of-flight mass spectrometer (Bruker Impact II, Bremen, Germany). Melting points were obtained on a SGW X-4 melting point apparatus. All solvents used were distilled with standard techniques.

2. Optimization of reaction conditions[a]

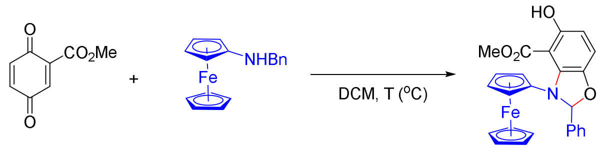

1a **2a** **3a**

entry	ratio	T (°C)	time (h)	yield (%)[b]
1	2:1	40	11	71
2	2:1	35	8	91
3	2:1	25	11	50
4	2:1	10	13	75
5	2:1	35	17	>99
6	2:1	35	24	95
7	3:1[c]	35	17	37
8	1:1[d]	35	17	40

[a] Unless otherwise noted, the reaction was carried out with **1a** (0.10 mmol, 2.0 equiv.), **2a** (0.05 mmol, 1.0 equiv.), DCM (0.5 mL). [b] Isolated yield. [c] 0.15 mmol **1a** (3.0 equiv.) was used. [d] 0.05 mmol **1a** (1.0 equiv.) was used. DCM = dichloromethane

3. General Procedure for Catalyst-free Aza-Michael Addition/C(sp^3)-O Bond Formation Tandem Reaction

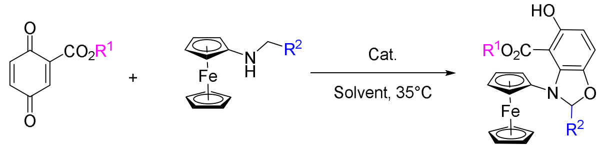

1 **2** **3**

p-Benzoquinones **1** (0.1 mmol, 1.0 equiv.), amino ferrocenes **2** (0.05 mmol, 0.5 equiv.) were dissolved in dichloromethane (0.5 mL) in a test tubes. The mixture was stirred at 35 °C in an oil bath and monitored by thin-layer chromatography (TLC). Upon the reaction completion, the mixture was charged onto a silica gel column directly, and the desired product **3** was purified by flash chromatography with petroleum ether/ethyl acetate (v/v = 15:1) as eluent.

3. Characterizations of Compounds **3**

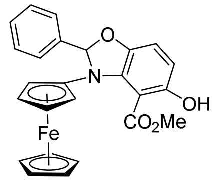

methyl 3-ferrocenyl-5-hydroxy-2-phenyl-2,3-dihydrobenzo[*d*]oxazole-4-carboxylate (3a). Brown oil. 22.6 mg, ＞99% yield. ^{1}H NMR (400 MHz, $CDCl_3$) δ 10.09 (s, 1H), 7.55 (dd, *J* = 7.5 Hz, *J* = 1.7 Hz, 2H), 7.40 (dd, *J* = 5.6 Hz, *J* = 3.7 Hz, 3H), 7.16 (s, 1H), 6.91 (d, *J* = 8.5 Hz, 1H), 6.49 (d, *J* = 8.6 Hz, 1H), 4.21 (s, 5H), 4.16 - 4.12 (m, 1H), 4.06 - 4.02 (m, 1H), 3.97 - 3.94 (m, 1H), 3.63 - 3.59 (m, 1H), 3.55 (s, 3H); ^{13}C NMR (101 MHz, $CDCl_3$) δ 169.3, 155.2, 143.9, 139.3, 135.8, 129.3, 128.7, 126.2, 114.6, 109.2, 108.2, 102.8, 102.5, 68.7, 64.9, 63.1, 62.1, 57.2, 51.8. HRMS (ESI-TOF) m/z: $[M]^+$ calcd for $C_{25}H_{21}FeNO_4$ 455.0814; found 455.0811.

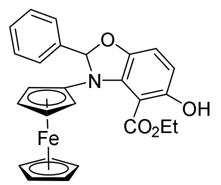

ethyl 3-ferrocenyl -5-hydroxy-2-phenyl-2,3-dihydrobenzo[*d*]oxazole-4-carboxylate (3b). Brown oil. 22.7 mg, 97% yield. ^{1}H NMR (400 MHz, $CDCl_3$) δ 10.18 (s, 1H), 7.53-7.46 (m, 2H), 7.36-7.26 (m, 3H), 7.08 (s, 1H), 6.86 (d, *J* = 8.6 Hz, 1H), 6.44 (d, *J* = 8.6 Hz, 1H), 4.13 (s, 5H), 4.10 (d, *J* = 2.1 Hz, 1H), 4.09-4.03 (m, 1H), 3.97-3.95 (m, 1H), 3.95-3.89 (m, 1H), 3.89-3.85 (m, 1H), 3.58-3.55 (m, 1H), 0.90 (t, *J* = 7.1 Hz, 3H); ^{13}C NMR (101 MHz, $CDCl_3$) δ 167.9, 154.3, 142.9, 138.4, 134.4, 128.1, 127.6, 125.1, 113.7, 108.6, 107.8, 102.0, 101.3, 67.7, 63.8, 62.0, 61.1, 60.3, 55.8, 12.5. HRMS (ESI-TOF) m/z: $[M]^+$ calcd for $C_{26}H_{23}FeNO_4$ 469.0971; found 469.0972.

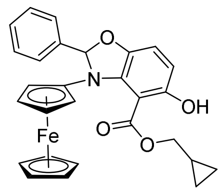

cyclopropylmethyl 3-ferrocenyl-5-hydroxy-2-phenyl-2,3-dihydrobenzo[*d*] oxazole-4-carboxylate (3c). Orange solid. 23.4 mg, 94% yield. m.p. = 120.5-122.1 °C. ^{1}H NMR (400 MHz, $CDCl_3$) δ 10.11 (s, 1H), 7.55 (dd, *J* = 7.4 Hz, *J* =1.7 Hz, 2H), 7.35-7.26 (m, 3H), 7.08 (s, 1H), 6.86 (d, *J* = 8.6 Hz, 1H), 6.44 (d, *J* = 8.6 Hz, 1H), 4.16-4.09 (m, 6H), 3.96-3.92 (m, 1H), 3.90-3.83 (m, 2H), 3.69 (dd, *J* = 11.3 Hz, *J* =7.0 Hz, 1H), 3.57 (dt, *J* = 2.6 Hz, *J* = 1.3 Hz, 1H), 0.73-0.62 (m, 1H), 0.44- 0.30 (m, 2H), 0.11-0.06 (m, 1H), 0.05-0.01 (m, 1H); ^{13}C NMR (101 MHz, $CDCl_3$) δ 167.9, 154.2, 142.9, 138.4, 134.3, 128.0, 127.5, 125.2, 113.7, 108.7, 107.6, 102.2, 100.9, 69.2, 67.6, 63.7, 62.1, 60.8, 55.8, 8.1, 2.32, 2.26. HRMS (ESI-TOF) m/z: $[M]^+$ calcd for $C_{28}H_{25}FeNO_4$ 495.1127; found 495.1122.

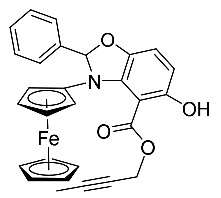

but-2-yn-1-yl 3-ferrocenyl-5-hydroxy-2-phenyl-2,3-dihydrobenzo[*d*]oxazole-4-carboxylate (3d). Orange oil. 20.0 mg, 81% yield. ^{1}H NMR (400 MHz, $CDCl_3$) δ 9.87 (s, 1H), 7.57-7.50 (m, 2H), 7.36-7.30 (m, 3H), 7.10 (s, 1H), 6.85 (d, *J* = 8.6 Hz, 1H), 6.42 (d, *J* = 8.6 Hz, 1H), 4.64 (dd, *J* = 15.1 Hz, *J* = 2.3 Hz, 1H), 4.26 (dd, *J* = 15.1 Hz, *J* = 2.3 Hz, 1H), 4.13 (s, 5H), 4.09 (d, *J* = 1.1 Hz, 1H), 3.97 (d, *J* = 1.2 Hz, 1H), 3.87 (d, *J* = 1.2 Hz, 1H), 3.57 (d, *J* = 1.1 Hz, 1H), 1.72 (t, *J* = 2.3 Hz, 3H); ^{13}C NMR (101 MHz, $CDCl_3$) δ 167.2, 158.3, 154.2, 143.0, 138.3, 134.8, 128.1, 127.6, 125.3, 113.8, 108.3, 107.3, 101.5, 101.3, 82.4, 71.4, 67.7, 63.8, 62.1, 61.1, 56.2, 52.2, 2.6. HRMS (ESI-TOF) m/z: $[M]^+$ calcd for $C_{28}H_{23}FeNO_4$ 493.0971; found 493.0969.

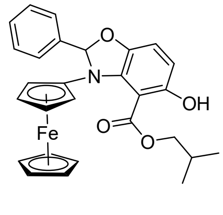

isobutyl 3-ferrocenyl-5-hydroxy-2-phenyl-2,3-dihydrobenzo[*d*]oxazole-4-carboxylate (3e). Orange solid. 14.8 mg, 59% yield. m.p. = 119.3-121.0 °C. ^{1}H NMR (400 MHz, $CDCl_3$) δ 10.22 (s, 1H), 7.51 (dd, *J* = 7.0 Hz, *J* = 2.1 Hz, 2H), 7.34-7.25 (m, 3H), 7.07 (s, 1H), 6.88 (d, *J* = 8.6 Hz, 1H), 6.47 (d, *J* = 8.6 Hz, 1H), 4.12 (s, 5H), 4.06 (d, *J* = 1.0 Hz, 1H), 3.94 (dd, *J* = 10.0 Hz, *J* = 8.2 Hz,

2H), 3.88-3.85 (m, 1H), 3.56 (d, J = 1.0 Hz, 1H), 3.52 (dd, J = 10.5 Hz, J = 5.7 Hz, 1H), 1.50 (d, J = 1.6 Hz, 1H), 0.70 (dd, J = 10.4 Hz, J = 6.7 Hz, 6H); ^{13}C NMR (101 MHz, $CDCl_3$) δ 168.2, 154.3, 142.9, 138.3, 134.3, 128.1, 127.5, 125.2, 113.8, 109.0, 107.6, 102.3, 101.2, 70.5, 67.6, 63.7, 62.1, 60.7, 55.9, 26.2, 18.2, 17.9. HRMS (ESI-TOF) m/z: $[M]^+$ calcd for $C_{28}H_{27}FeNO_4$ 497.1284; found 497.1280.

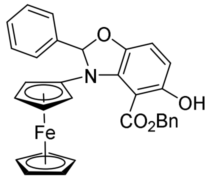

benzyl 3-ferrocenyl-5-hydroxy-2-phenyl-2,3-dihydrobenzo[*d*]oxazole-4-carboxylate (3f). Orange oil. 11.2 mg, 42% yield. ^{1}H NMR (400 MHz, $CDCl_3$) δ 9.98 (s, 1H), 7.34-7.26 (m, 4H), 7.26-7.19 (m, 4H), 7.17-7.14 (m, 1H), 7.03 (dd, J = 7.6 Hz, J = 1.4 Hz, 2H), 7.00 (s, 1H), 6.86 (d, J = 8.6 Hz, 1H), 6.44 (d, J = 8.6 Hz, 1H), 5.22 (s, 1H), 4.64 (d, J = 11.8 Hz, 1H), 4.10 (s, 5H), 3.90 (dd, J = 3.8, 2.5 Hz, 1H), 3.87 (dd, J = 3.8 Hz, J = 2.4 Hz, 1H), 3.43-3.38 (m, 1H); ^{13}C NMR (101 MHz, $CDCl_3$) δ 167.5, 154.1, 143.0, 138.3, 134.4, 133.4, 128.0, 127.9, 127.6, 127.4, 127.3, 127.2, 126.8, 126.3, 125.1, 113.8, 108.6, 107.4, 102.0, 100.9, 67.6, 66.1, 63.8, 62.2, 60.6, 56.0. HRMS (ESI-TOF) m/z: $[M]^+$ calcd for $C_{31}H_{25}FeNO_4$ 531.1127; found 531.1124.

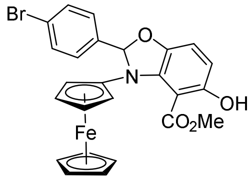

methyl 2-(4-bromophenyl)-3-ferrocenyl-5-hydroxy-2,3-dihydrobenzo[*d*] oxazole-4-carboxylate (3g). Brown solid. 13.4 mg, 50% yield. m.p. = 197.9-199.5 °C. ^{1}H NMR (400 MHz, $CDCl_3$) δ 10.02 (s, 1H), 7.46 (d, J = 8.4 Hz, 2H), 7.36 (d, J = 8.4 Hz, 2H), 7.02 (s, 1H), 6.85 (d, J = 8.6 Hz, 1H), 6.44 (d, J = 8.6 Hz, 1H), 4.13 (s, 5H), 4.07-4.03 (m, 1H), 4.01-3.95 (m, 1H), 3.89 (d, J = 1.3 Hz, 1H), 3.55-3.50 (m, 1H), 3.48 (s, 3H); ^{13}C NMR (101 MHz, $CDCl_3$) δ 168.2, 154.4, 142.7, 137.4, 134.4, 131.4, 130.9, 130.0, 127.0, 122.3, 113.8, 108.6, 107.2, 101.7, 101.0, 67.7, 63.9, 62.1, 61.1, 56.1, 50.8. HRMS (ESI-TOF) m/z: $[M]^+$ calcd for $C_{25}H_{20}BrFeNO_4$ 532.9920; found 532.9917.

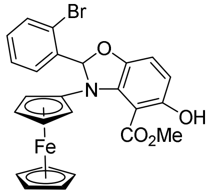

methyl 2-(2-bromophenyl)-3-ferrocenyl-5-hydroxy-2,3-dihydrobenzo[*d*] oxazole-4-carboxylate (3h). Orange solid. 18.0 mg, 68% yield. m.p. = 170.1-171.8 °C. ^{1}H NMR (400 MHz, $CDCl_3$) δ 10.01 (s, 1H), 7.69 (dd, J = 7.4 Hz, J =1.6 Hz, 1H), 7.61 (s, 1H), 7.48 (dd, J = 7.2 Hz, J =2.2 Hz, 1H), 7.28 (dd, J = 5.5 Hz, J =1.9 Hz, 2H), 6.89 (d, J = 8.6 Hz, 1H), 6.49 (d, J = 8.6 Hz, 1H), 4.27 (s, 5H), 4.21-4.17 (m, 1H), 4.02 (dd, J = 3.7 Hz, J =2.4 Hz, 1H), 3.98-3.94 (m, 1H), 3.55 (s, 3H), 3.49- 3.45 (m, 1H); ^{13}C NMR (101 MHz, $CDCl_3$) δ 169.2, 155.1, 143.2, 137.3, 136.1, 133.3, 131.0, 128.3, 128.0, 122.8, 114.8, 109.2, 108.0, 103.0, 102.0, 68.9, 65.0, 63.0, 62.4, 57.6, 51.8. HRMS (ESI-TOF) m/z: $[M]^+$ calcd for $C_{25}H_{20}BrFeNO_4$ 532.9920; found 532.9912.

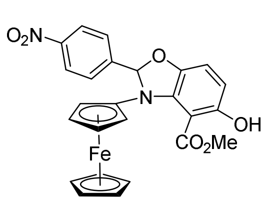

methyl 3-ferrocenyl-5-hydroxy-2-(4-nitrophenyl)-2,3-dihydrobenzo[*d*] oxazole-4-carboxylate (3i). Brown solid. 11.7 mg, 47% yield. m.p. = 180.2-182.0 °C. ^{1}H NMR (400 MHz, $CDCl_3$) δ 10.11 (s, 1H), 8.23-8.15 (m, 2H), 7.68 (d, J = 8.6 Hz, 2H), 7.11 (s, 1H), 6.91 (d, J = 8.6 Hz, 1H), 6.49 (d, J = 8.6 Hz, 1H), 4.14 (s, 5H), 4.05-4.04 (m, 1H), 4.02-4.00 (m, 1H), 3.92-3.91 (m, 1H), 3.58-3.56 (m, 1H), 3.52 (s, 3H); ^{13}C NMR (101 MHz, $CDCl_3$) δ 168.0, 154.7, 147.4, 145.3, 142.5, 133.9, 129.5, 126.3, 123.3, 122.9, 114.2, 109.5, 107.5, 102.2, 100.3, 67.8, 64.0, 62.3, 61.1, 55.9, 50.9. HRMS (ESI-TOF) m/z: $[M]^+$ calcd for $C_{25}H_{20}FeN_2O_6$ 500.0665; found 500.0653.

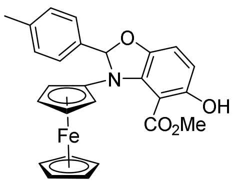

methyl 3-ferrocenyl-5-hydroxy-2-(p-tolyl)-2,3-dihydrobenzo[*d*]oxazole-4-carboxylate (3j). Orange oil. 12.3 mg, 52% yield. ^{1}H NMR (400 MHz, $CDCl_3$) δ 10.00 (s, 1H), 7.36 (d, *J* = 8.0 Hz, 2H), 7.14 (d, *J* = 7.9 Hz, 2H), 7.06 (s, 1H), 6.82 (d, *J* = 8.5 Hz, 1H), 6.41 (d, *J* = 8.5 Hz, 1H), 4.13 (s, 5H), 4.09-4.05 (m, 1H), 3.96 (d, *J* = 1.3 Hz, 1H), 3.88 (d, *J* = 1.3 Hz, 1H), 3.54-3.50 (m, 1H), 3.46 (s, 3H), 2.29 (s, 3H); ^{13}C NMR (101 MHz, $CDCl_3$) δ 168.3, 154.0, 142.9, 138.2, 135.4, 134.9, 128.4, 125.1, 113.4, 107.9, 107.1, 101.8, 101.3, 67.7, 63.8, 62.0, 61.1, 56.2, 50.7, 20.2. HRMS (ESI-TOF) m/z: $[M]^+$ calcd for $C_{26}H_{23}FeNO_4$ 469.0971; found 469.0972.

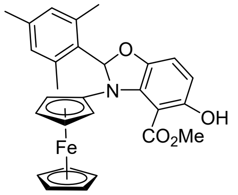

methyl 3-ferrocenyl-5-hydroxy-2-mesityl-2,3-dihydrobenzo[*d*]oxazole-4-carboxylate (3k). Yellow solid. 16.4 mg, 66% yield. m.p. = 166.1-167.8 °C. ^{1}H NMR (400 MHz, $CDCl_3$) δ 9.79 (s, 1H), 7.60 (s, 1H), 6.82 (s, 2H), 6.75 (d, *J* = 8.5 Hz, 1H), 6.38 (d, *J* = 8.5 Hz, 1H), 4.19-4.11 (m, 6H), 3.94-3.86 (m, 2H), 3.42 (s, 3H), 3.23-3.18 (m, 1H), 2.38 (s, 6H), 2.22 (s, 3H); ^{13}C NMR (101 MHz, $CDCl_3$) δ 168.4, 153.2, 144.2, 138.0, 136.6, 135.1, 130.4, 129.7, 111.7, 106.9, 106.5, 101.1, 100.8, 67.5, 63.7, 61.6, 60.6, 56.3, 50.5, 19.9, 19.5. HRMS (ESI-TOF) m/z: $[M]^+$ calcd for $C_{28}H_{27}FeNO_4$ 497.1284; found 497.1282.

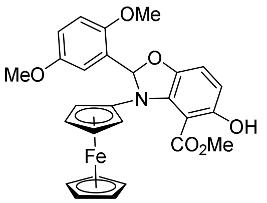

methyl 3-ferrocenyl-2-(2,5-dimethoxyphenyl)-5-hydroxy-2,3-dihydrobenzo[*d*]oxazole-4-carboxylate (3l). Orange solid. 16.8 mg, 65% yield. m.p. = 93.7-95.5 °C. ^{1}H NMR (400 MHz, $CDCl_3$) δ 9.87 (s, 1H), 7.49 (s, 1H), 6.95 (d, *J* = 3.0 Hz, 1H), 6.87 (d, *J* = 8.9 Hz, 1H), 6.84-6.81 (m, 1H), 6.79 (s, 1H), 6.38 (d, *J* = 8.5 Hz, 1H), 4.15 (s, 5H), 4.12-4.09 (m, 1H), 3.95-3.92 (m, 1H), 3.91 (s, 3H), 3.87 (dd, *J* = 3.7 Hz, *J* = 2.4 Hz, 1H), 3.61 (s, 3H), 3.49-3.46 (m, 1H), 3.45 (s, 3H); ^{13}C NMR (101 MHz, $CDCl_3$) δ 168.3, 153.7, 152.7, 150.4, 142.8, 135.2, 126.6, 114.0, 113.3, 112.4, 111.2, 107.5, 106.7, 100.8, 98.1, 67.8, 63.8, 62.0, 61.2, 56.7, 55.5, 54.5, 50.7. HRMS (ESI-TOF) m/z: $[M]^+$ calcd for $C_{27}H_{25}FeNO_6$ 515.1026; found 515.1019.

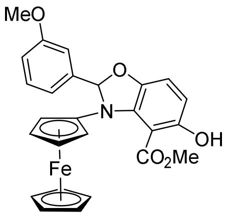

methyl 3-ferrocenyl-5-hydroxy-2-(3-methoxyphenyl)-2,3-dihydrobenzo[*d*] oxazole-4-carboxylate (3m). Orange solid. 18.3 mg, 75% yield. m.p. = 108.6-110.4 °C. ^{1}H NMR (400 MHz, $CDCl_3$) δ 9.99 (s, 1H), 7.25 (t, *J* = 7.9 Hz, 1H), 7.08 (d, *J* = 7.7 Hz, 1H), 7.05 (s, 1H), 7.03 (s, 1H), 6.84 (dd, *J* = 8.3 Hz, *J* = 2.9 Hz, 2H), 6.42 (d, *J* = 8.6 Hz, 1H), 4.12 (d, *J* = 3.8 Hz, 5H), 4.06 (dd, *J* = 2.4 Hz, *J* = 1.3 Hz, 1H), 3.98-3.95 (m, 1H), 3.90-3.86 (m, 1H), 3.73 (s, 3H), 3.57-3.54 (m, 1H), 3.47 (s, 3H); ^{13}C NMR (101 MHz, $CDCl_3$) δ 169.3, 159.9, 155.2, 143.9, 140.9, 135.7, 129.9, 118.5, 114.6, 111.8, 109.2, 108.2, 102.5, 68.7, 64.9, 63.1, 62.1, 57.2, 55.2, 51.7. HRMS (ESI-TOF) m/z: $[M]^+$ calcd for $C_{26}H_{23}FeNO_5$ 485.0920; found 485.0914.

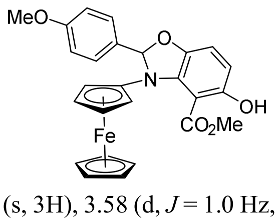

methyl 3-ferrocenyl-5-hydroxy-2-(4-methoxyphenyl)-2,3-dihydro-benzo[*d*]oxazole-4-carboxylate (3n). Orange oil. 15.7 mg, 65% yield. ^{1}H NMR (400 MHz, $CDCl_3$) δ 10.06 (s, 1H), 7.47 (d, *J* = 8.6 Hz, 2H), 7.12 (s, 1H), 6.93 (d, *J* = 8.7 Hz, 2H), 6.89 (d, *J* = 8.5 Hz, 1H), 6.48 (d, *J* = 8.5 Hz, 1H), 4.21 (s, 5H), 4.14 (s, 1H), 4.04 (s, 1H), 3.95 (d, *J* = 1.2 Hz, 1H), 3.81 (s, 3H), 3.58 (d, *J* = 1.0 Hz, 1H), 3.54 (s, 3H); ^{13}C NMR (101 MHz, $CDCl_3$) δ 169.4, 160.4, 155.0, 143.9, 138.4, 135.9, 131.5, 128.1, 127.6, 114.4, 114.1, 108.9, 108.0, 102.7, 102.2, 68.7, 64.8, 63.0, 62.1, 57.2, 55.4, 51.8. HRMS (ESI-TOF) m/z: $[M]^+$ calcd for $C_{26}H_{23}FeNO_5$ 485.0920; found 485.0913.

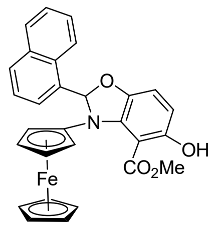

benzyl 3-ferrocenyl-5-hydroxy-2-(naphthalen-1-yl)-2,3-dihydrobenzo[*d*] oxazole-4-carboxylate (3o). Orange solid. 23.2 mg, 92% yield. m.p. = 129.6-131.3 °C. ^{1}H NMR (400 MHz, $CDCl_3$) δ 9.97 (s, 1H), 8.33 (d, J = 8.4 Hz, 1H), 7.87 (d, J = 7.7 Hz, 2H), 7.81 (d, J = 8.2 Hz, 1H), 7.65 (dd, J = 12.6 Hz, J = 4.0 Hz, 2H), 7.53 (t, J = 7.5 Hz, 1H), 7.40-7.32 (m, 1H), 6.76 (d, J = 8.5 Hz, 1H), 6.39 (d, J = 8.5 Hz, 1H), 4.20-4.18 (m, 1H), 4.17 (s, 5H), 3.94-3.88 (m, 2H), 3.55 (s, 3H), 3.44-3.41 (m, 1H); ^{13}C NMR (101 MHz, $CDCl_3$) δ 168.3, 154.0, 142.8, 135.0, 133.1, 132.5, 129.9, 129.0, 127.9, 125.9, 125.0, 124.4, 123.1, 122.7, 113.8, 108.1, 107.4, 101.3, 100.7, 67.8, 63.9, 62.1, 61.3, 56.4, 50.8. HRMS (ESI-TOF) m/z: $[M]^+$ calcd for $C_{29}H_{23}FeNO_4$ 505.0971; found 505.0972.

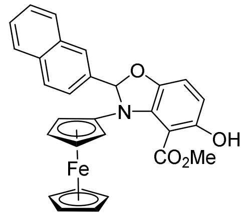

methyl 3-ferrocenyl-5-hydroxy-2-(naphthalen-2-yl)-2,3-dihydrobenzo[*d*] oxazole-4-carboxylate (3p). Orange oil. 18.5 mg, 73% yield. ^{1}H NMR (400 MHz, $CDCl_3$) δ 10.02 (s, 1H), 7.93 (s, 1H), 7.82 (d, J = 8.6 Hz, 1H), 7.80-7.74 (m, 2H), 7.57 (dd, J = 8.5 Hz, J = 1.6 Hz, 1H), 7.43 (dd, J = 6.2 Hz, J = 3.2 Hz, 2H), 7.24 (s, 1H), 6.86 (d, J = 8.5 Hz, 1H), 6.43 (d, J = 8.6 Hz, 1H), 4.17 (s, 5H), 4.11-4.10 (m, 1H), 3.98-3.96 (m, 1H), 3.91-3.90 (m, 1H), 3.58-3.57 (m, 1H), 3.50 (s, 3H); ^{13}C NMR (101 MHz, $CDCl_3$) δ 168.3, 154.1, 143.0, 135.5, 134.8, 132.7, 131.9, 127.8, 127.4, 126.7, 125.6, 125.4, 124.5, 122.8, 113.5, 108.1, 107.1, 102.0, 101.4, 67.7, 63.9, 62.0, 61.1, 56.3, 50.8. HRMS (ESI-TOF) m/z: $[M]^+$ calcd for $C_{29}H_{23}FeNO_4$ 505.0971; found 505.0973.

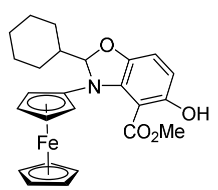

methyl 2-cyclohexyl-3-ferrocenyl-5-hydroxy-2,3-dihydrobenzo[*d*]oxazole-4-carboxylate (3q). Orange oil. 4.5 mg, 19% yield. ^{1}H NMR (400 MHz, $CDCl_3$) δ 10.13 (s, 1H), 6.85 (d, J = 8.5 Hz, 1H), 6.45 (d, J = 8.5 Hz, 1H), 5.79 (d, J = 6.2 Hz, 1H), 4.05 (s, 5H), 3.95 (d, J = 1.2 Hz, 1H), 3.89 (d, J = 1.0 Hz, 1H), 3.81 (d, J = 1.2 Hz, 1H), 3.60 (s, 1H), 3.47 (s, 3H), 1.76-1.55 (m, 6H), 1.16-1.03 (m, 5H); ^{13}C NMR (101 MHz, $CDCl_3$) δ 168.5, 153.9, 144.3, 135.5, 113.1, 108.5, 108.4, 106.4, 102.3, 67.6, 63.6, 61.9, 60.8, 56.1, 50.7, 42.4, 26.7, 26.3, 25.3, 24.8, 24.6. HRMS (ESI-TOF) m/z: $[M]^+$ calcd for $C_{25}H_{27}FeNO_4$ 461.1284; found 461.1284.

5. NMR Spectra for compounds **3**

^{1}H NMR of **3a**

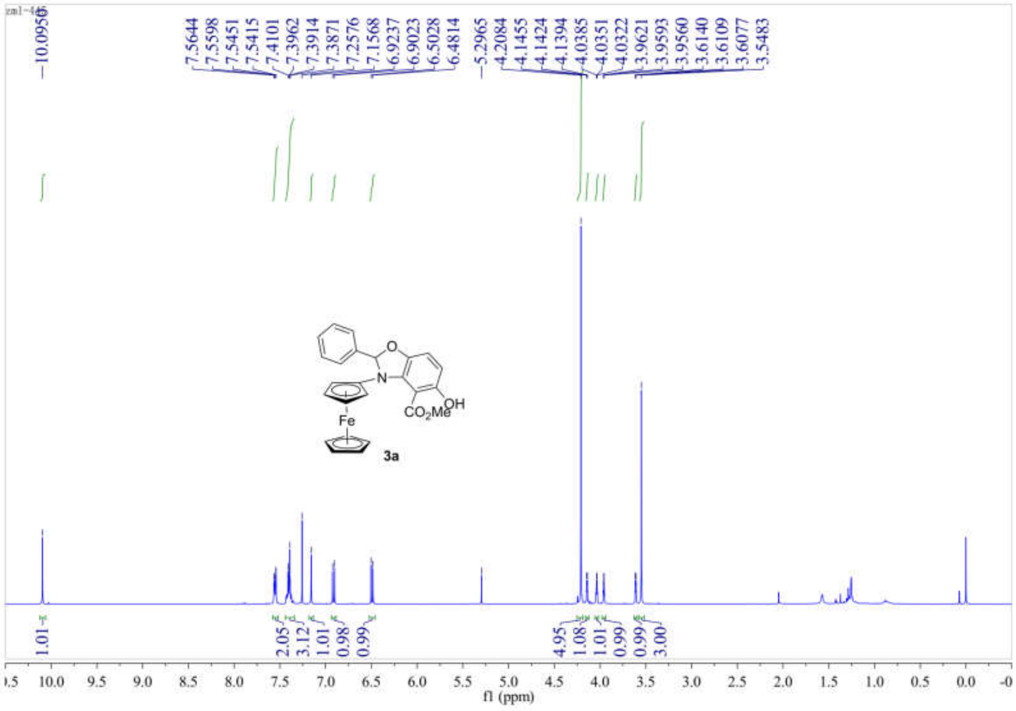

^{13}C NMR of **3a**

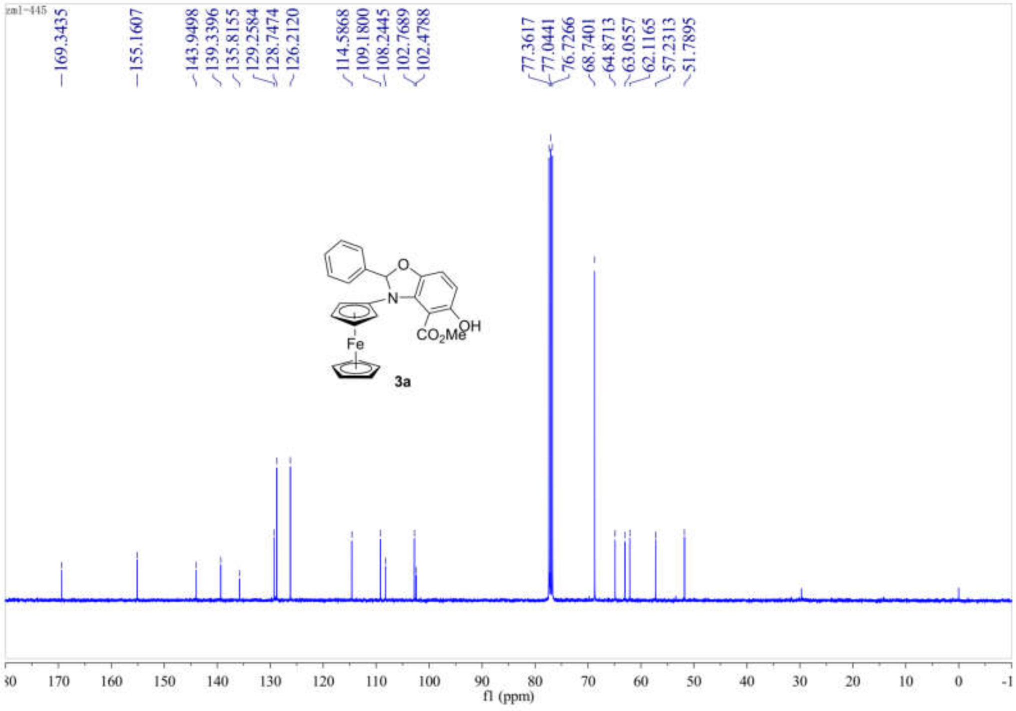

^{1}H NMR of **3b**

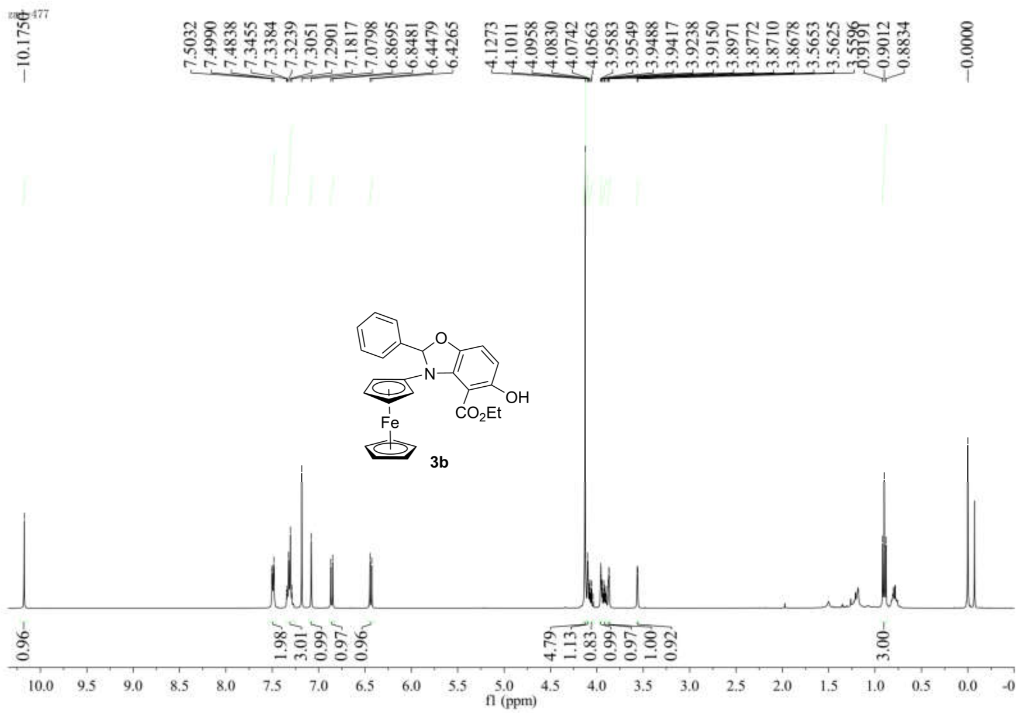

^{13}C NMR of **3b**

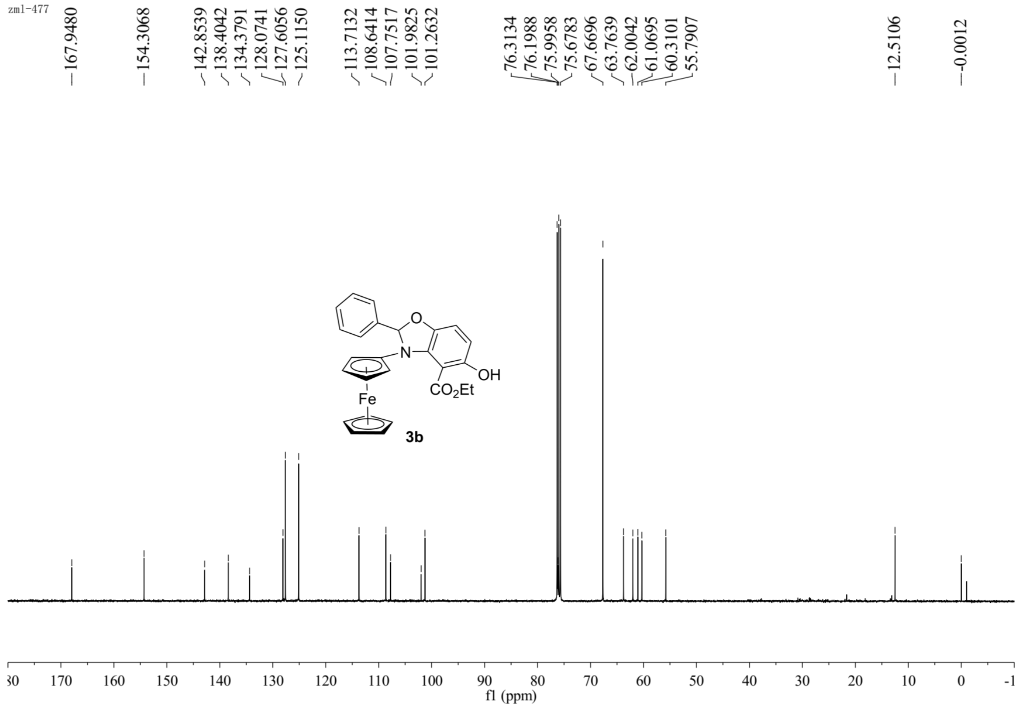

^{1}H NMR of **3c**

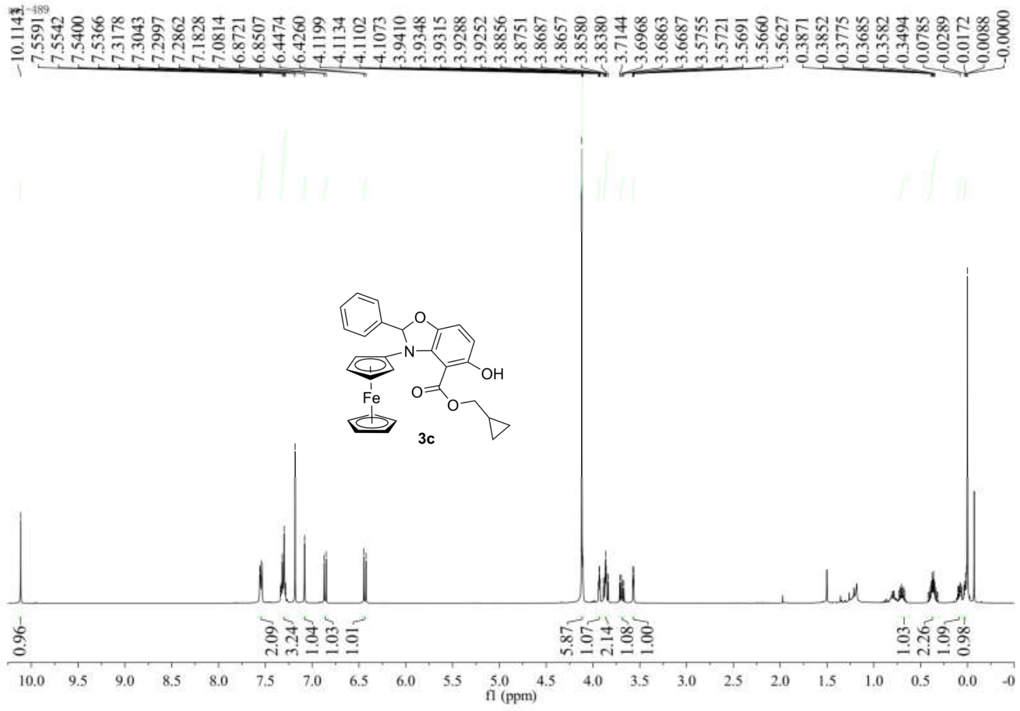

^{13}C NMR of **3c**

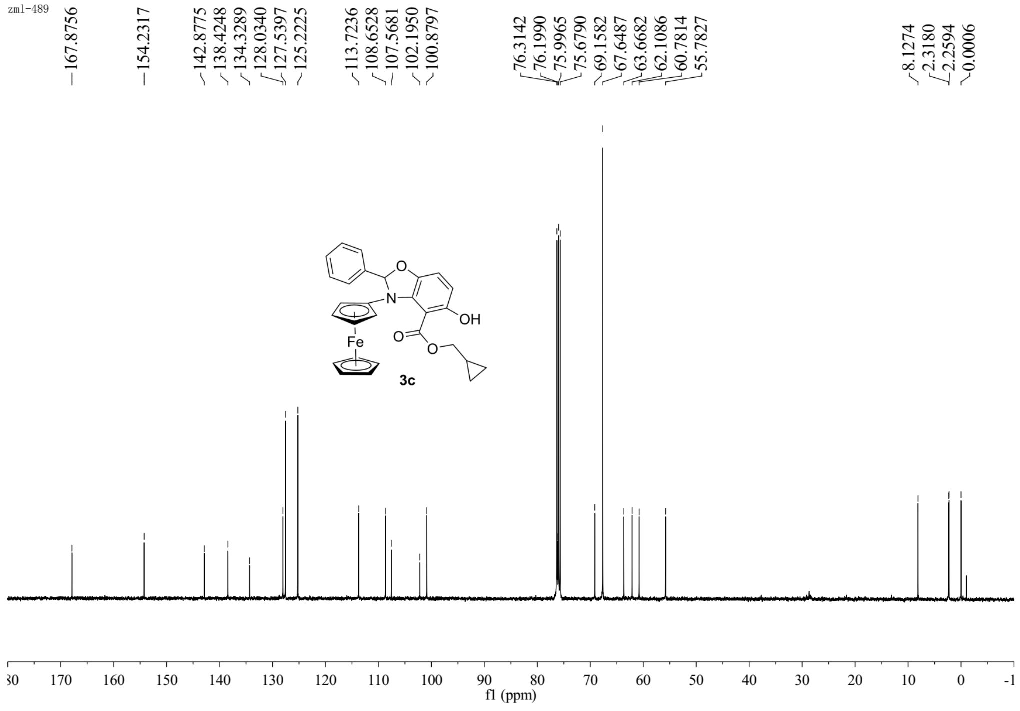

^{1}H NMR of **3d**

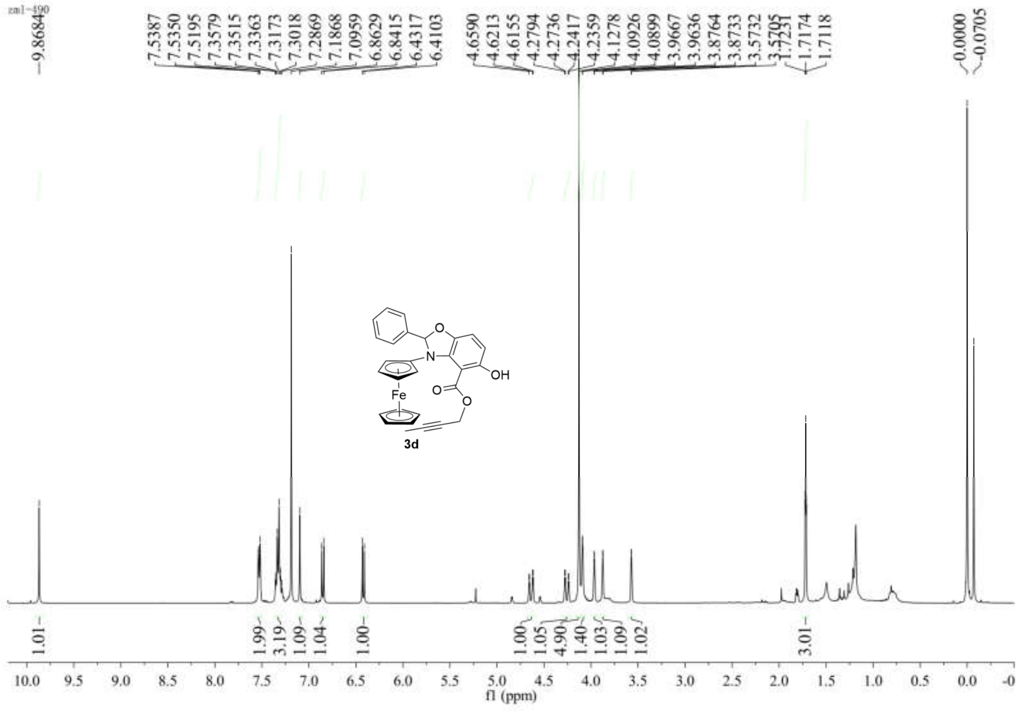

^{13}C NMR of **3d**

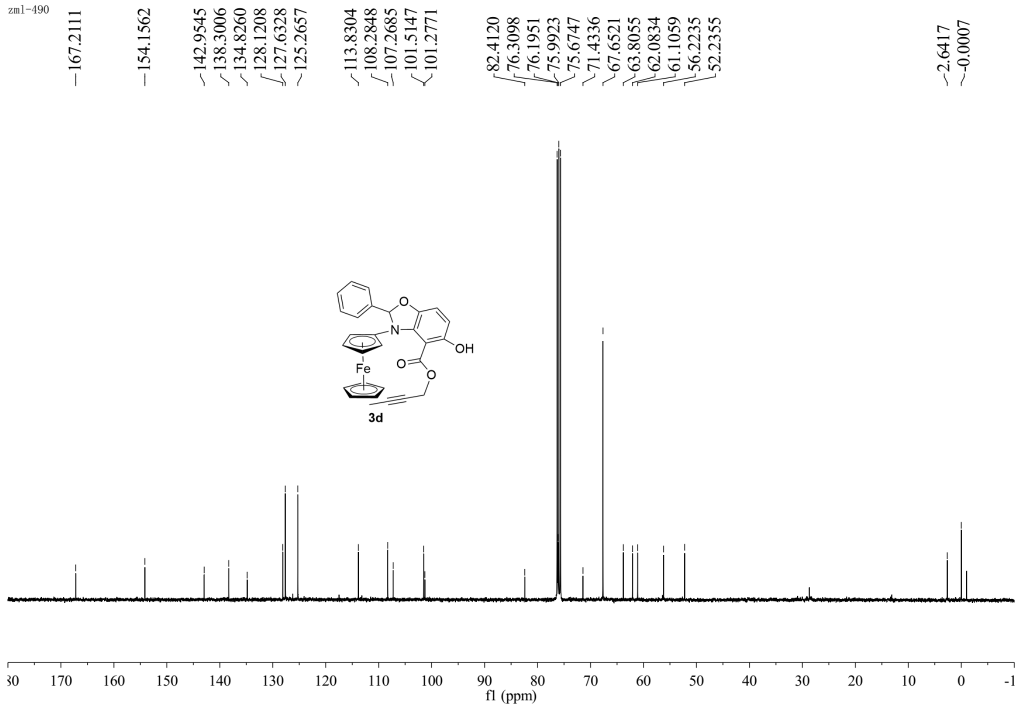

^{1}H NMR of **3e**

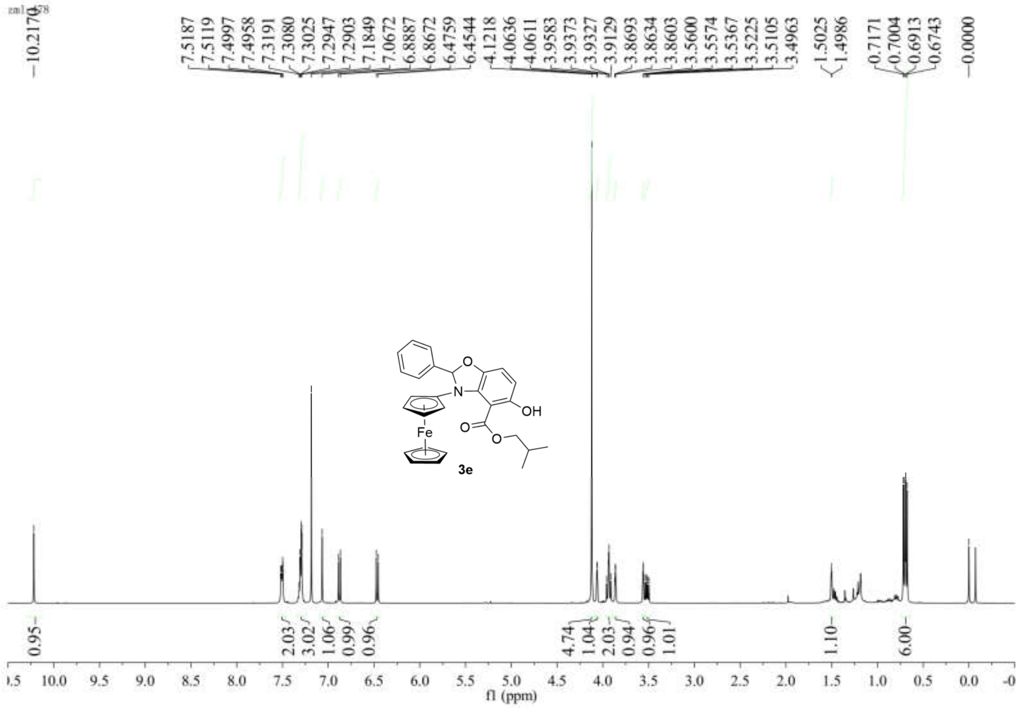

^{13}C NMR of **3e**

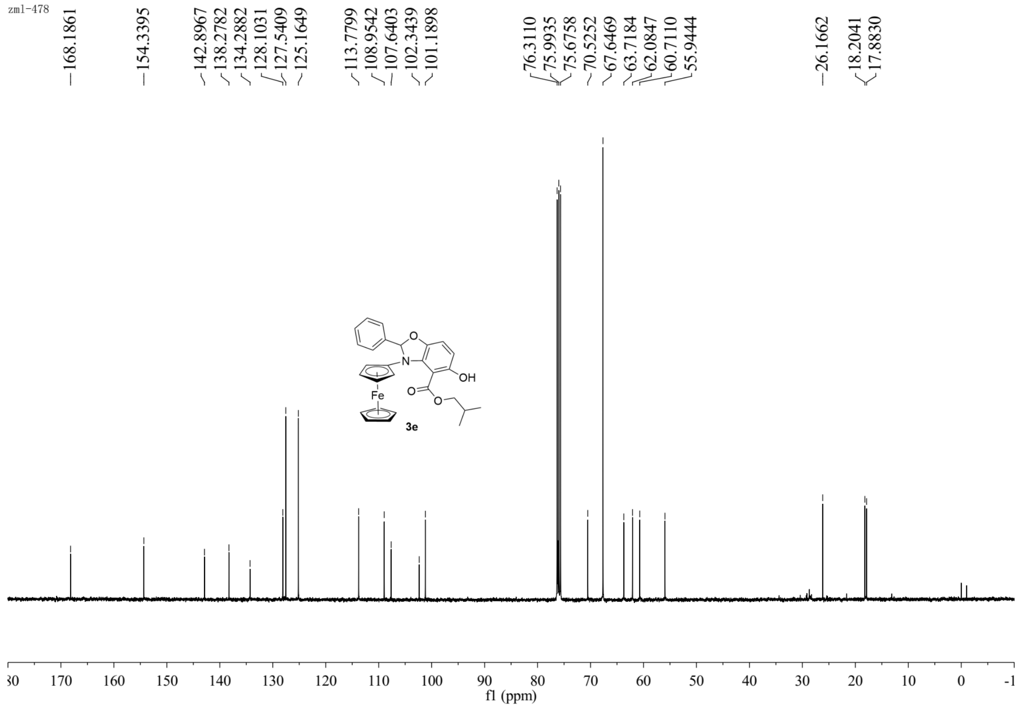

^{1}H NMR of **3f**

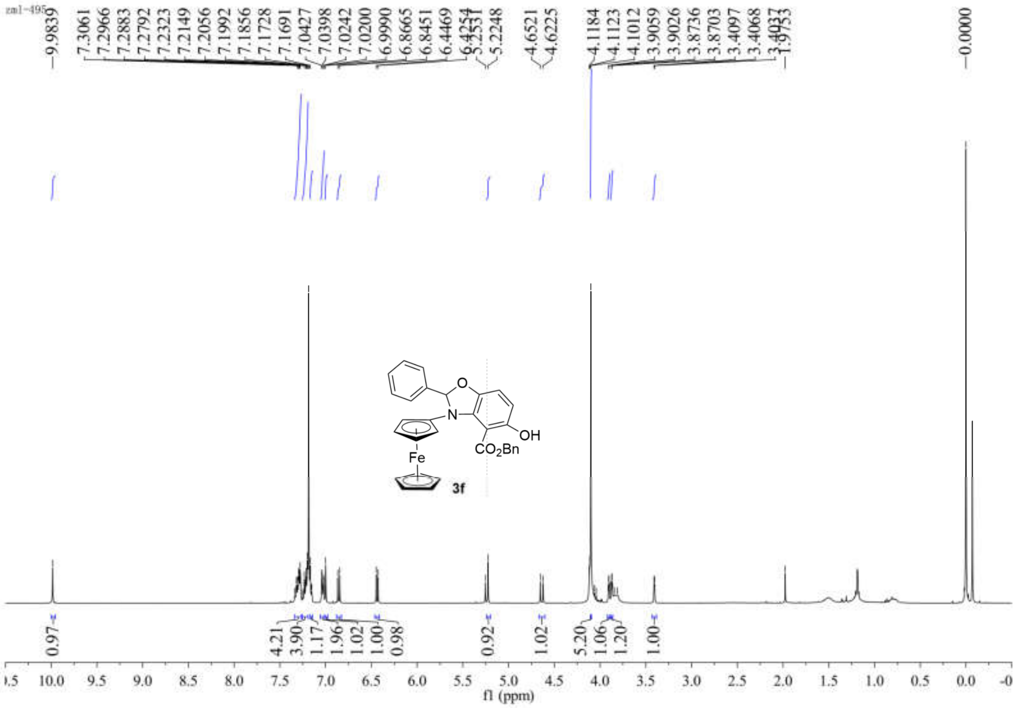

^{13}C NMR of **3f**

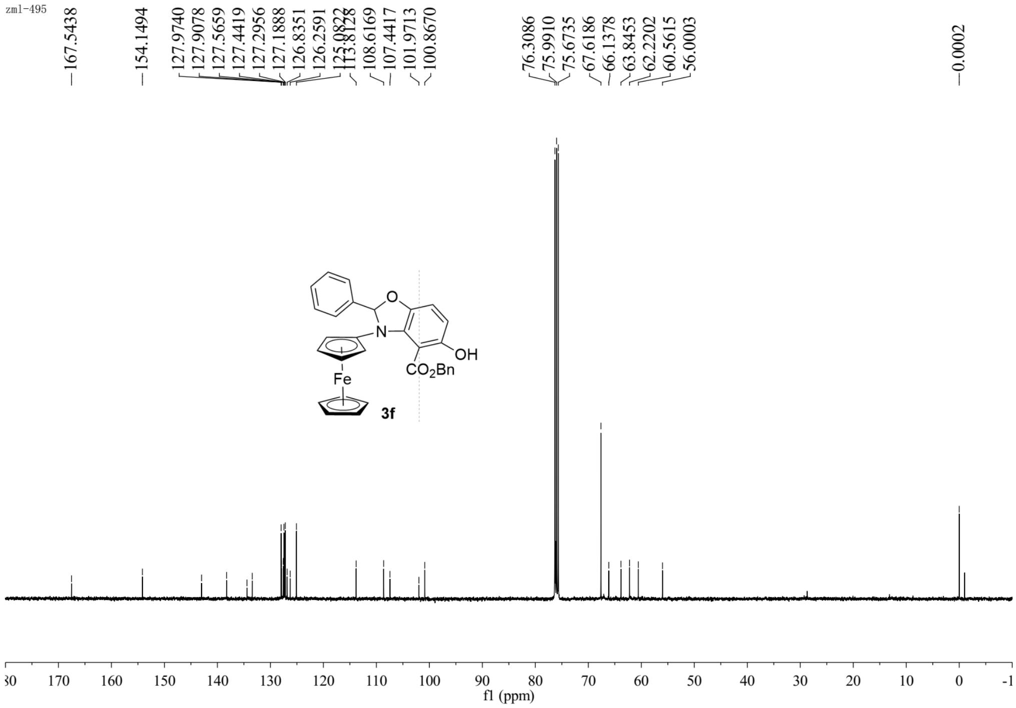

^{1}H NMR of **3g**

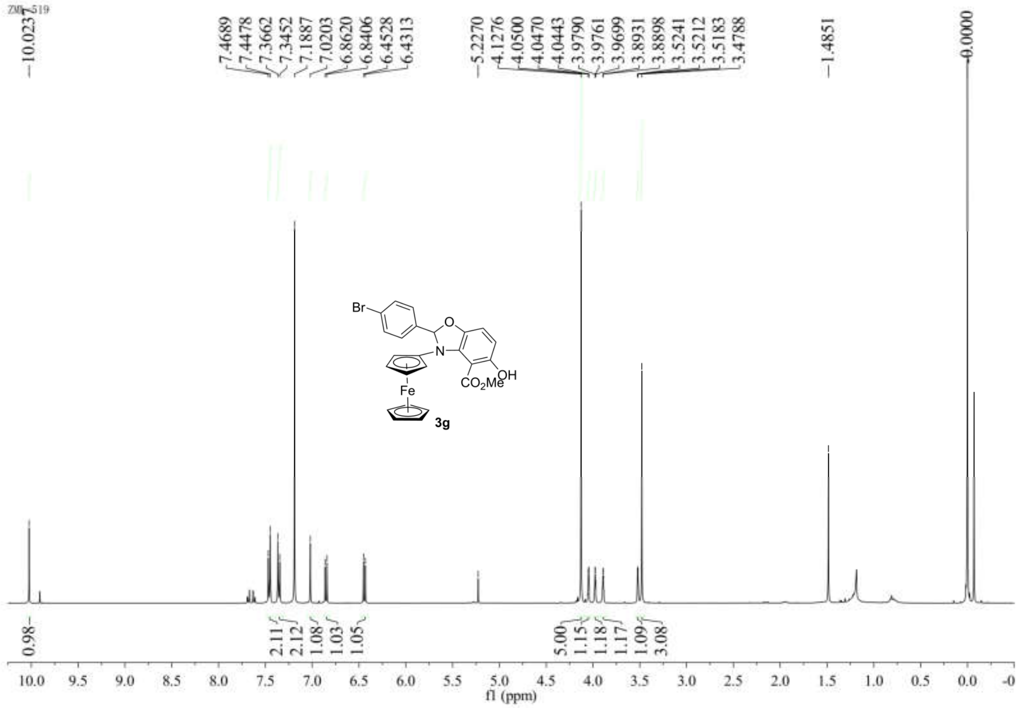

^{13}C NMR of **3g**

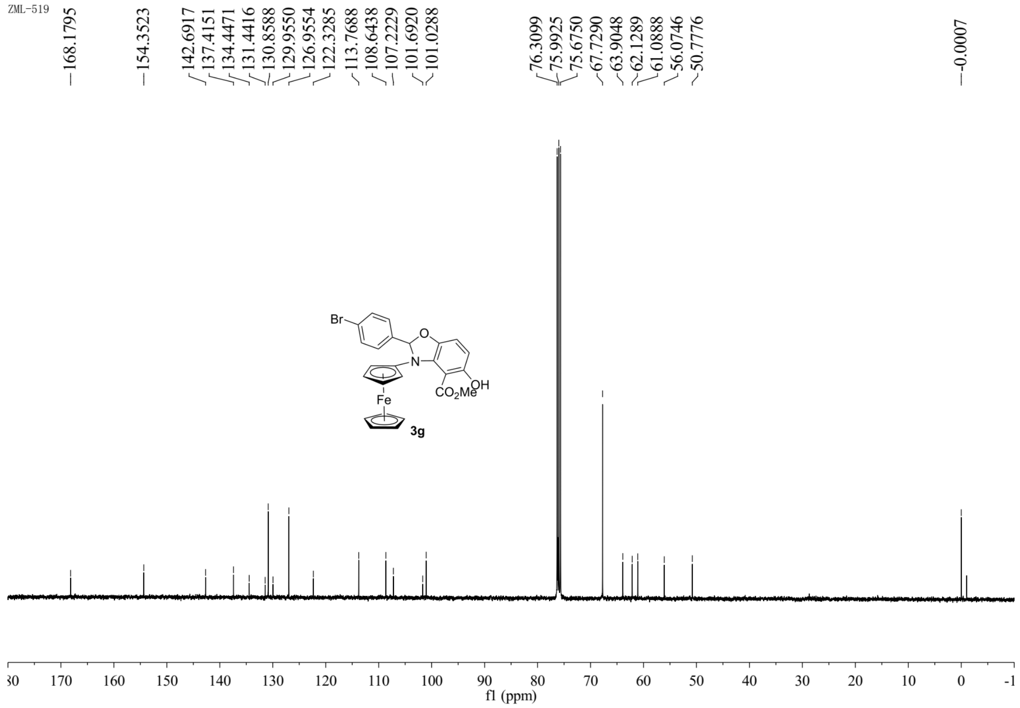

^{1}H NMR of **3h**

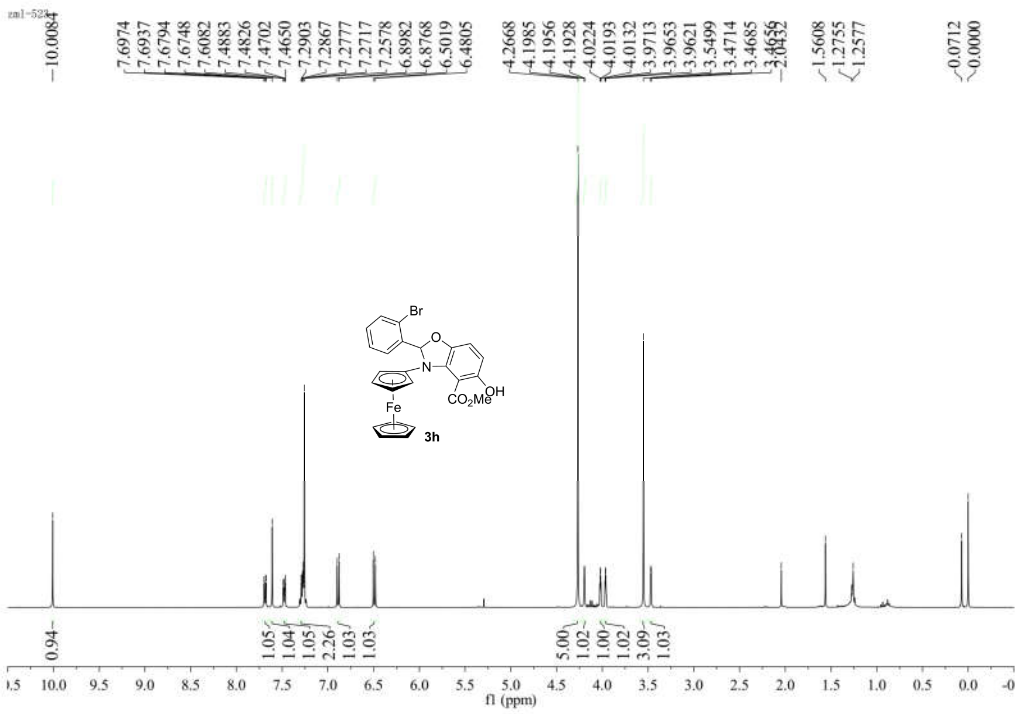

^{13}C NMR of **3h**

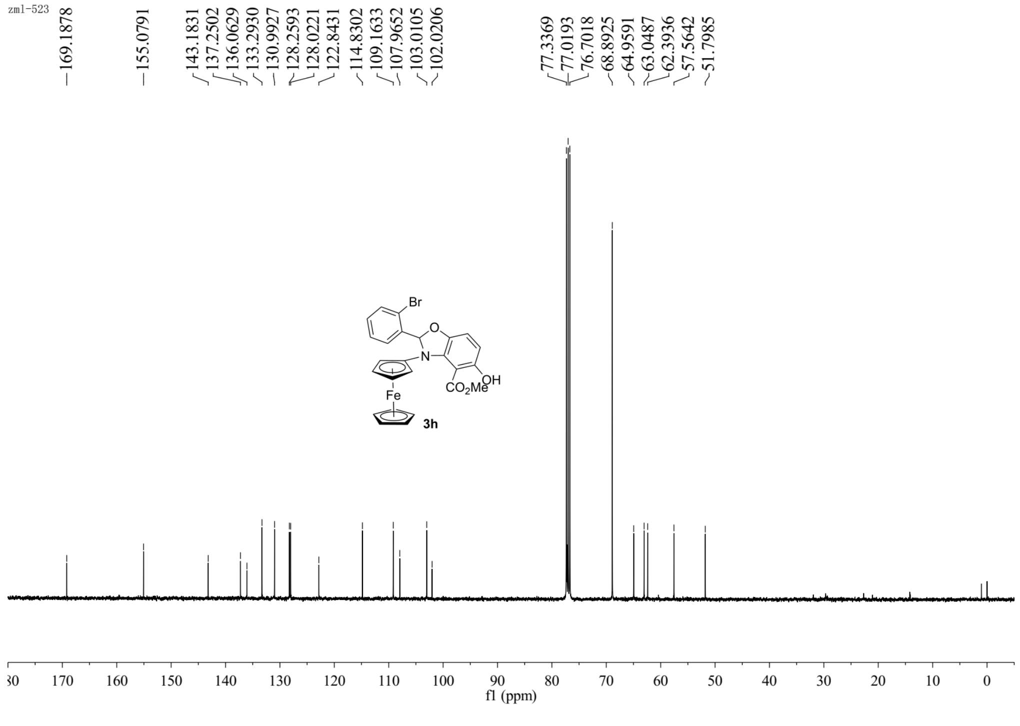

^{1}H NMR of **3i**

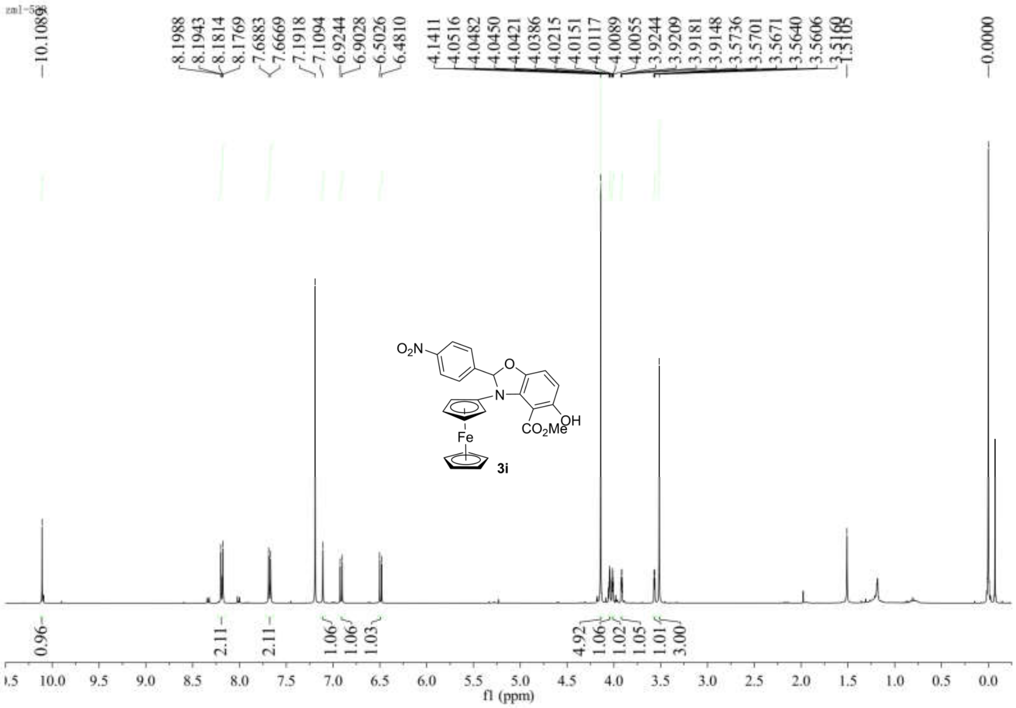

^{13}C NMR of **3i**

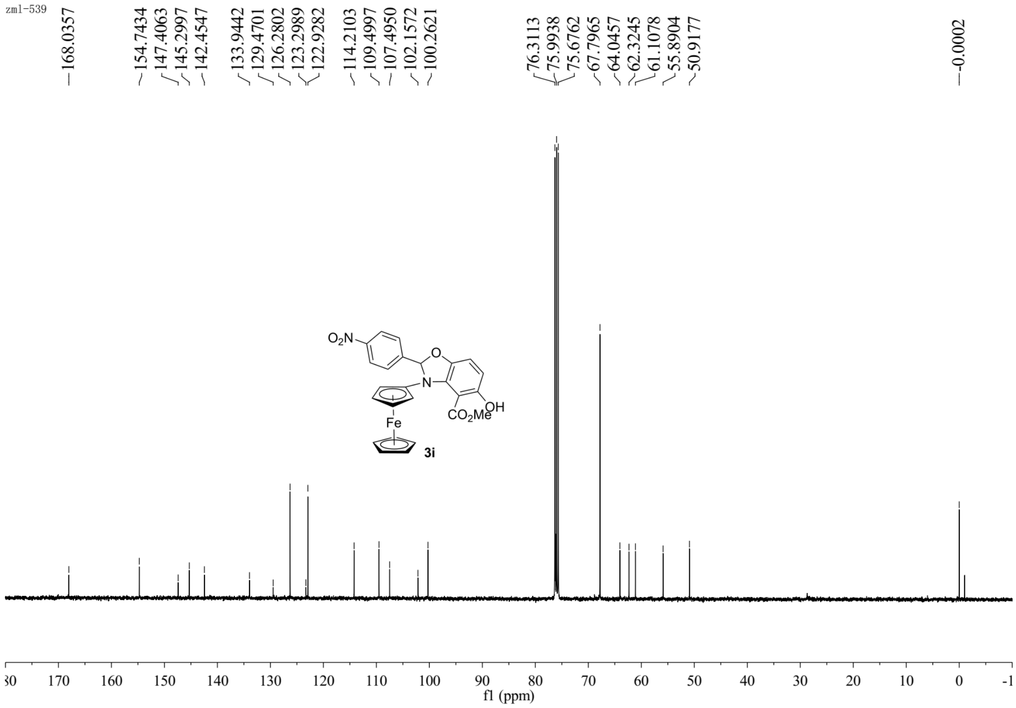

^{1}H NMR of **3j**

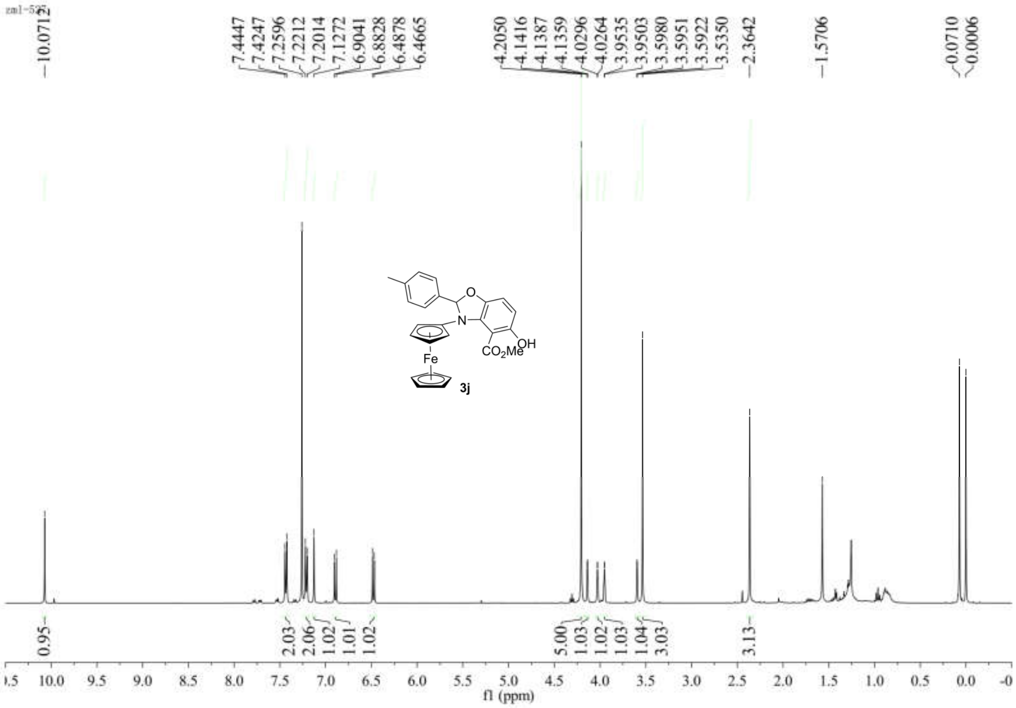

^{13}C NMR of **3j**

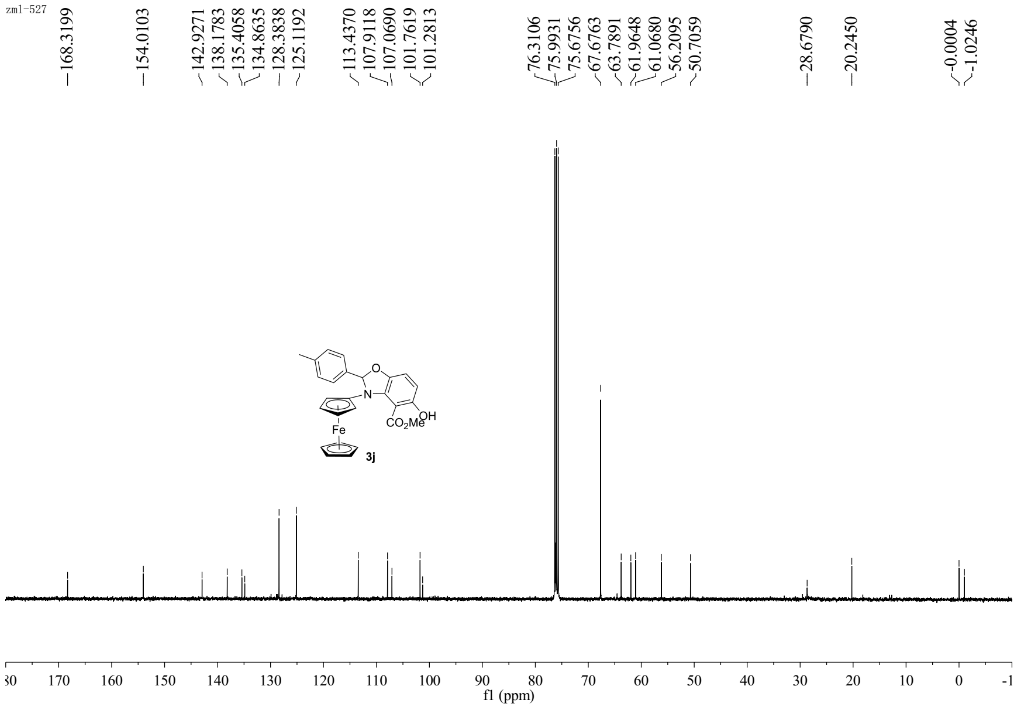

^{1}H NMR of **3k**

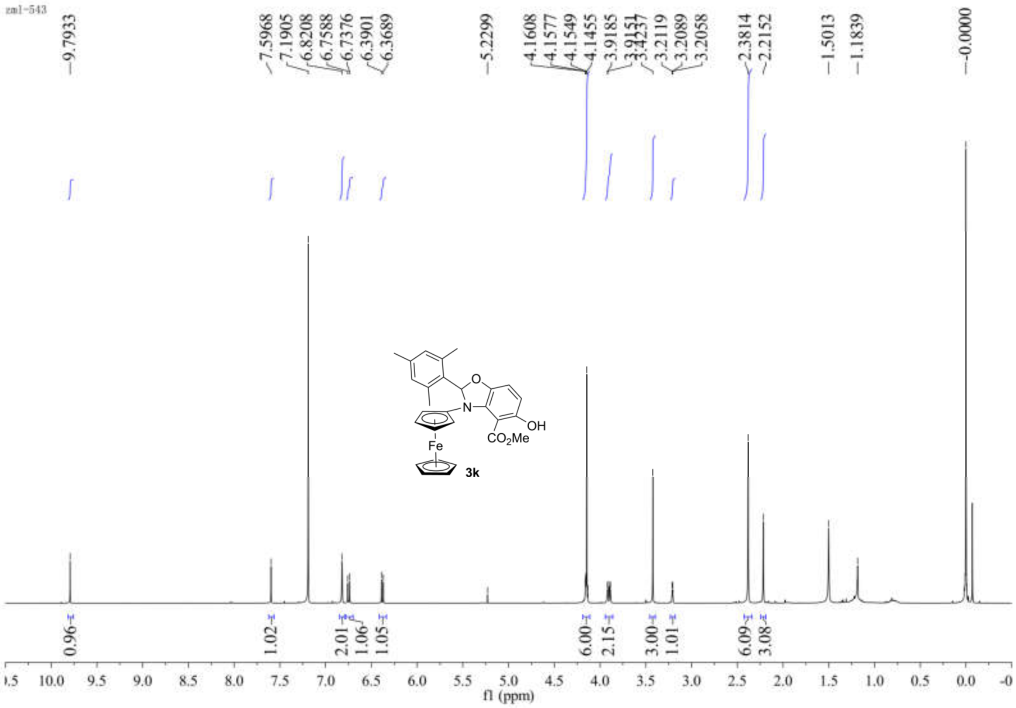

^{13}C NMR of **3k**

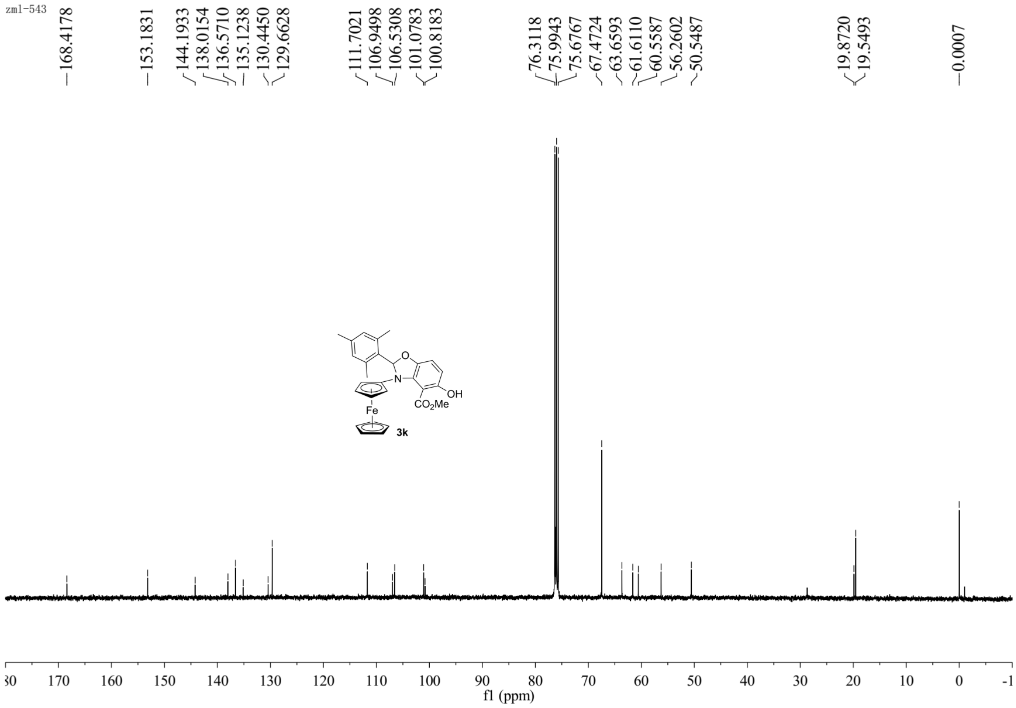

^{1}H NMR of **3l**

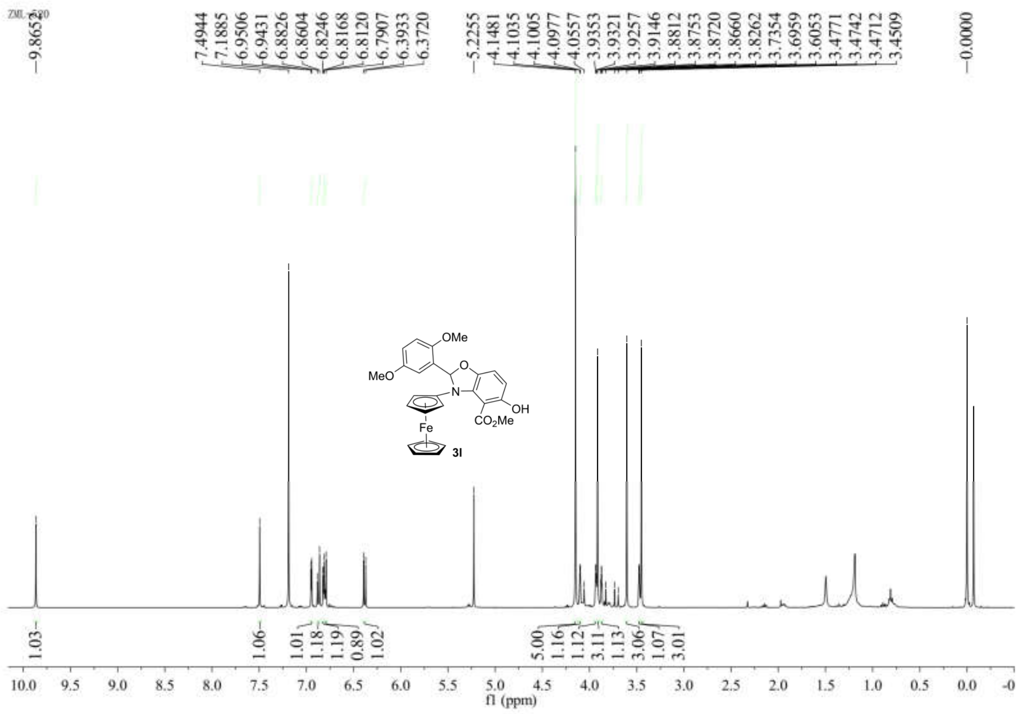

^{13}C NMR of **3l**

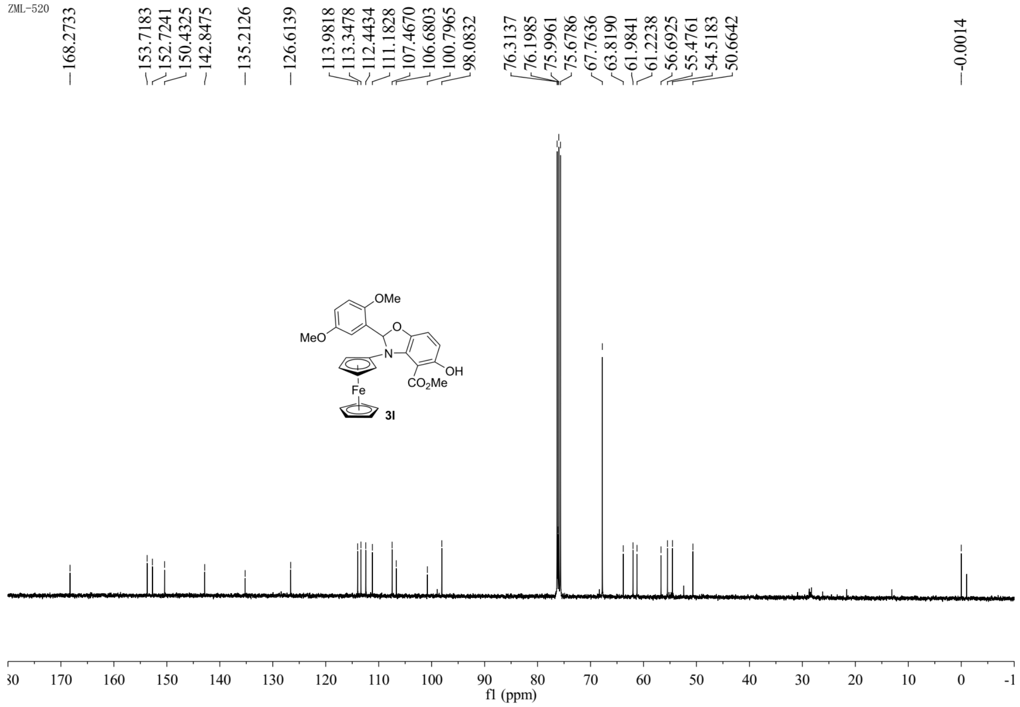

^{1}H NMR of **3m**

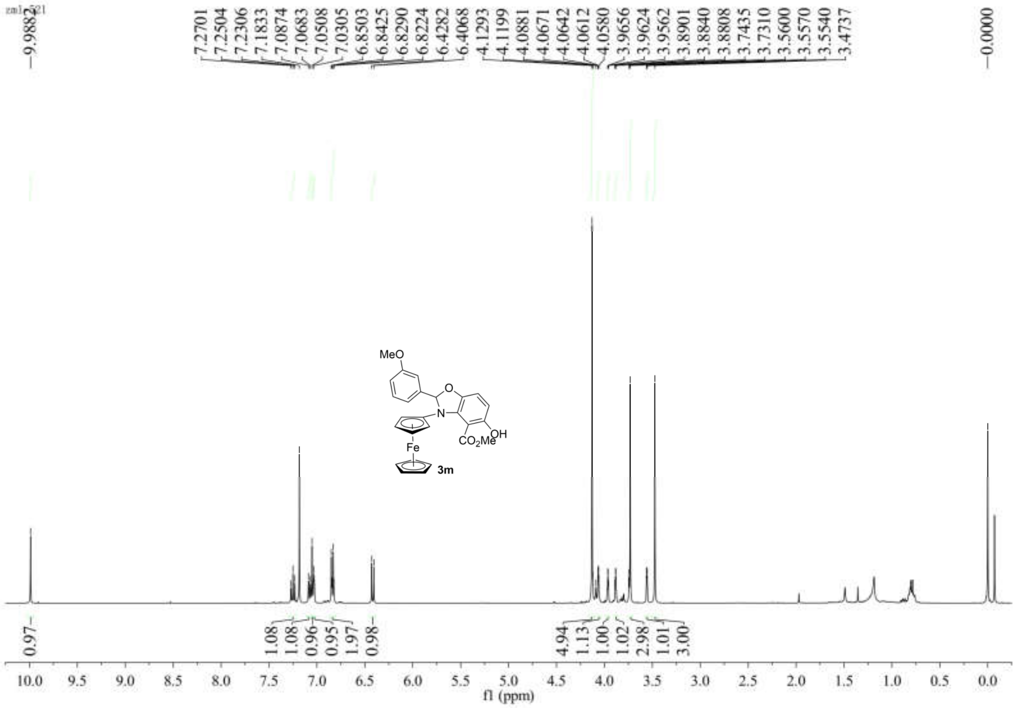

^{13}C NMR of **3m**

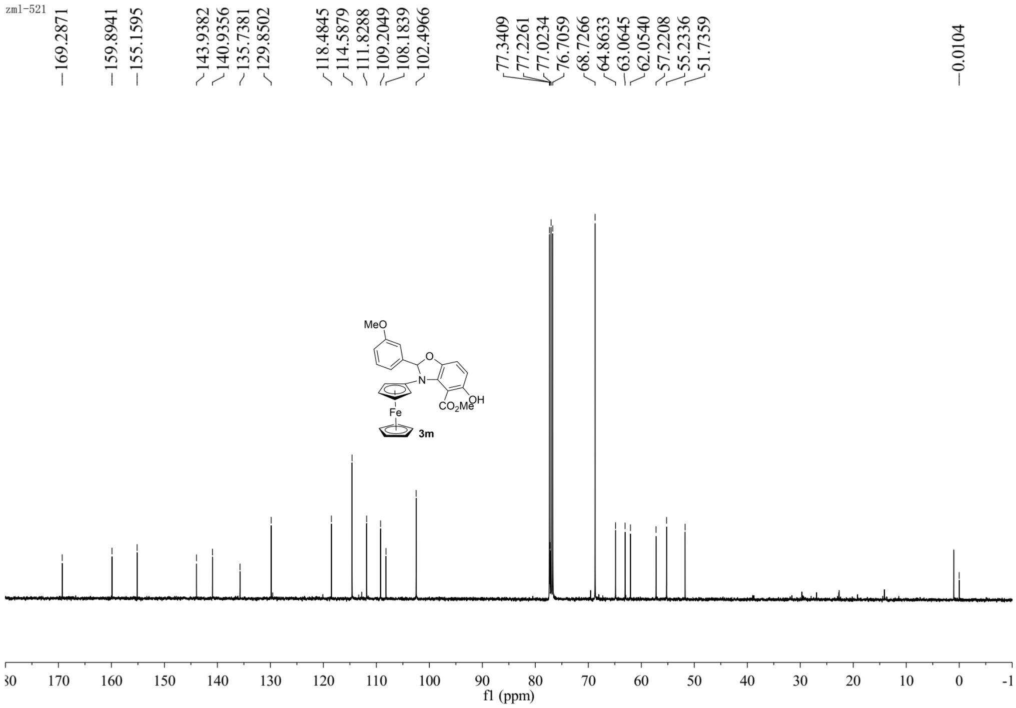

^{1}H NMR of **3n**

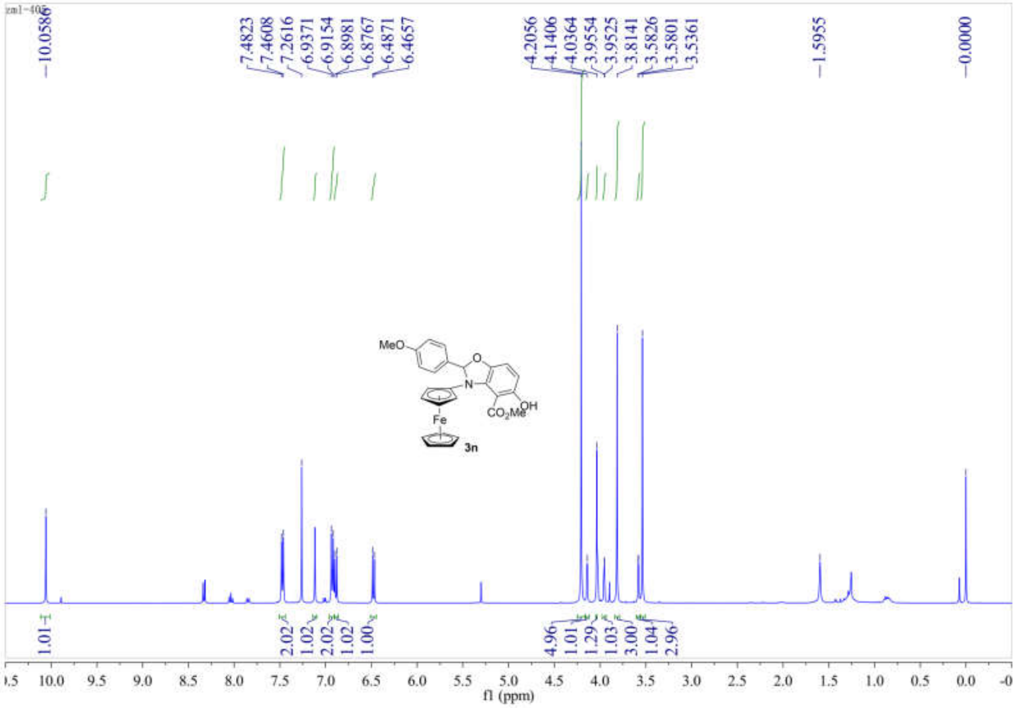

^{13}C NMR of **3n**

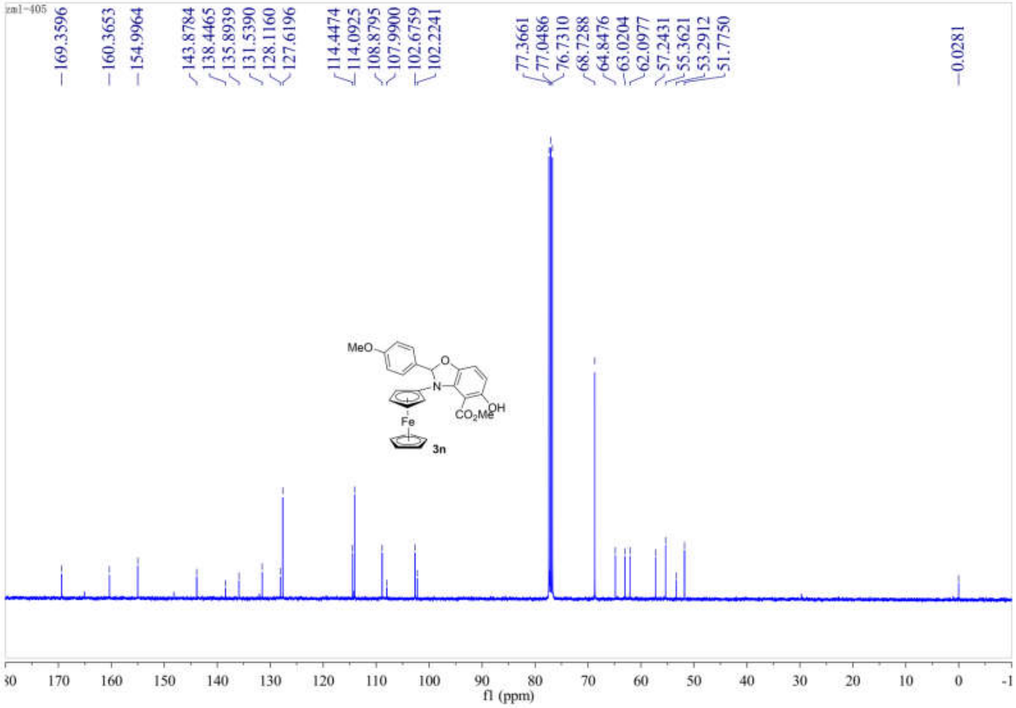

^{1}H NMR of **3o**

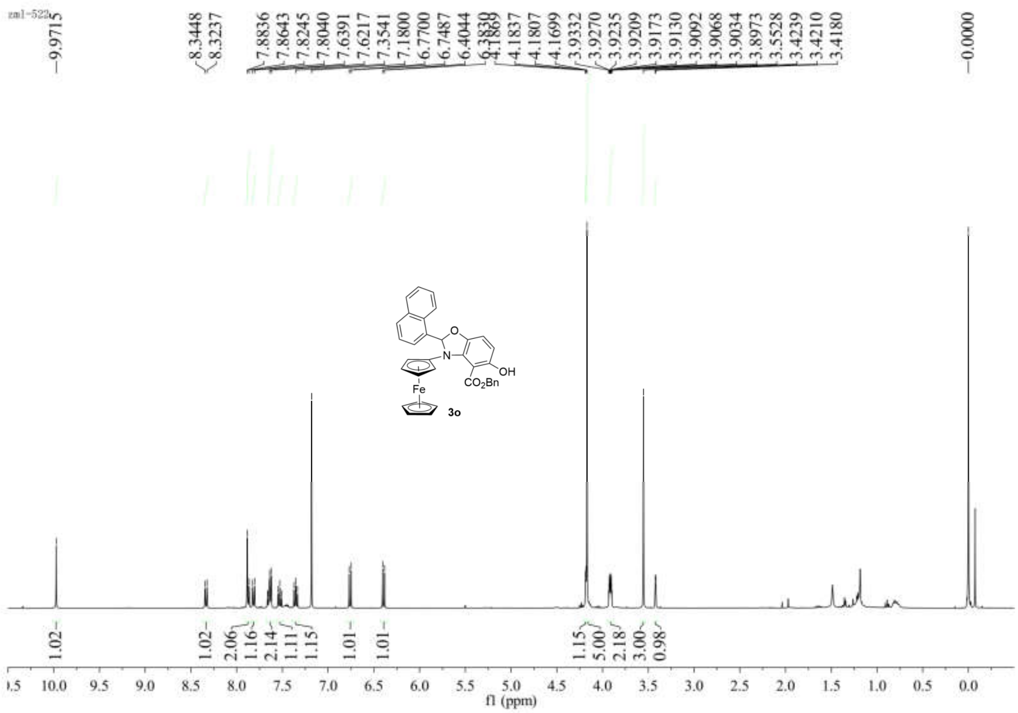

^{13}C NMR of **3o**

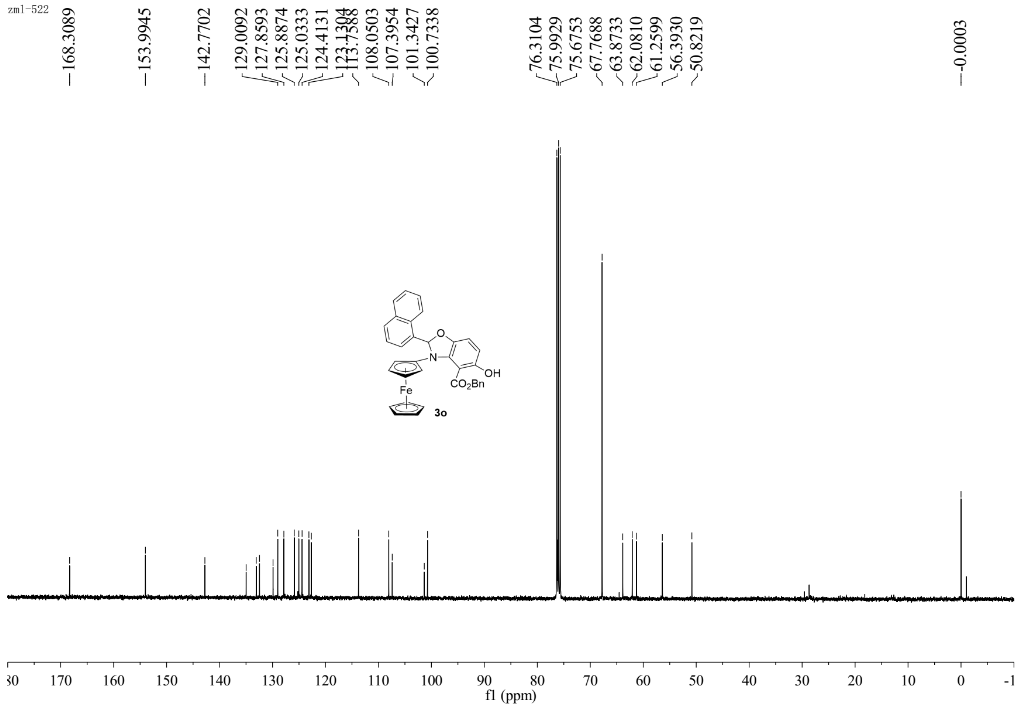

^{1}H NMR of **3p**

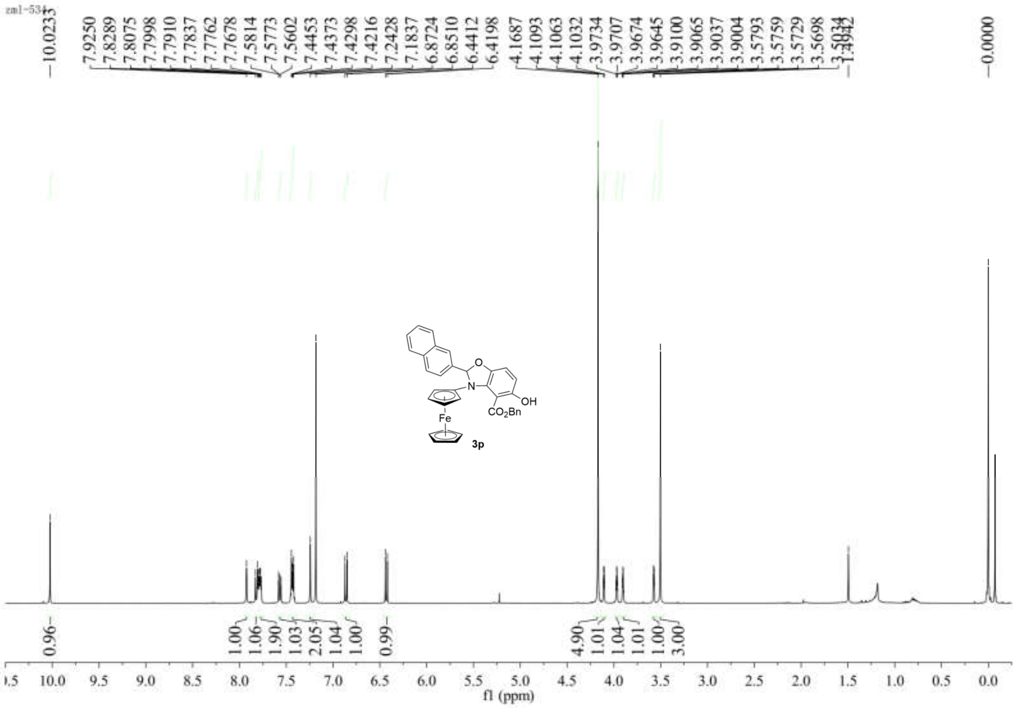

^{13}C NMR of **3p**

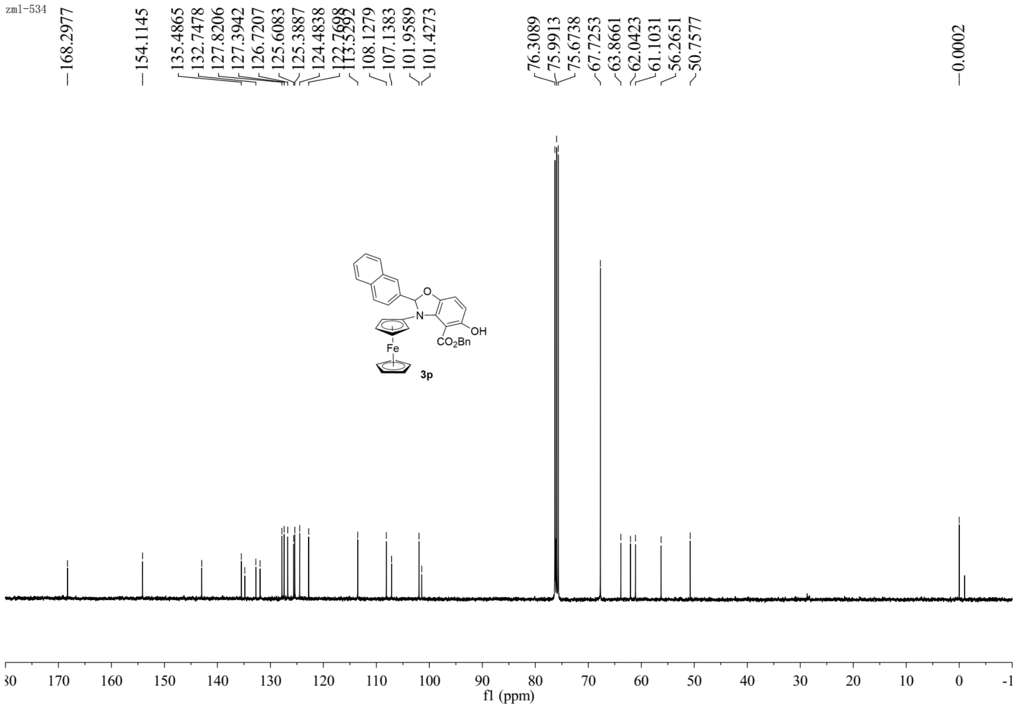

^{1}H NMR of **3q**

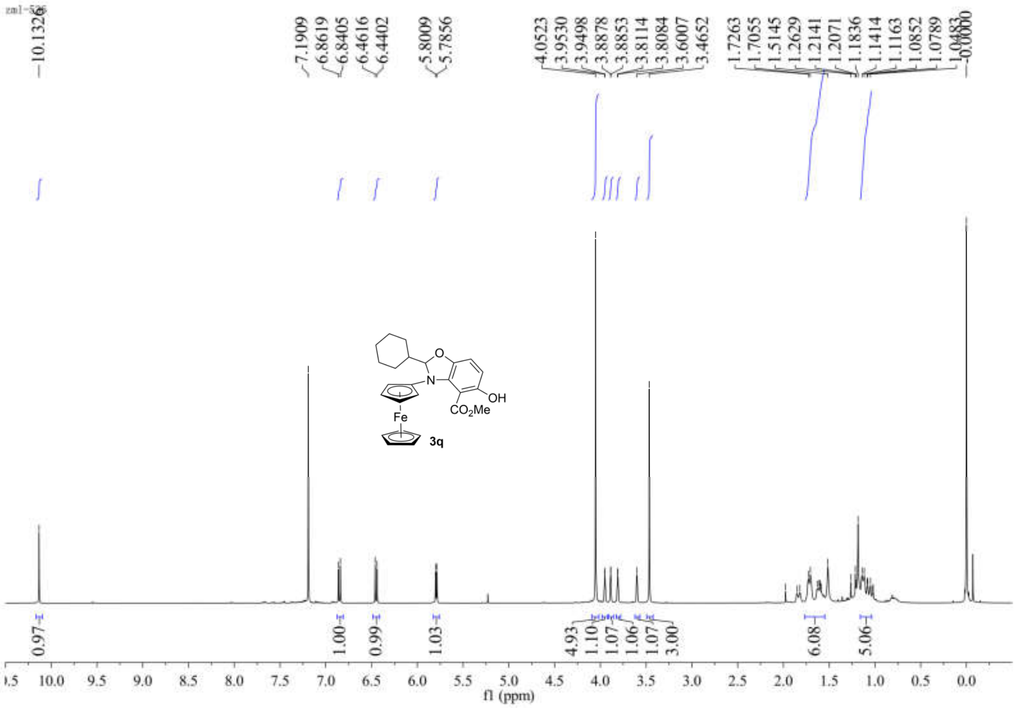

^{13}C NMR of **3q**

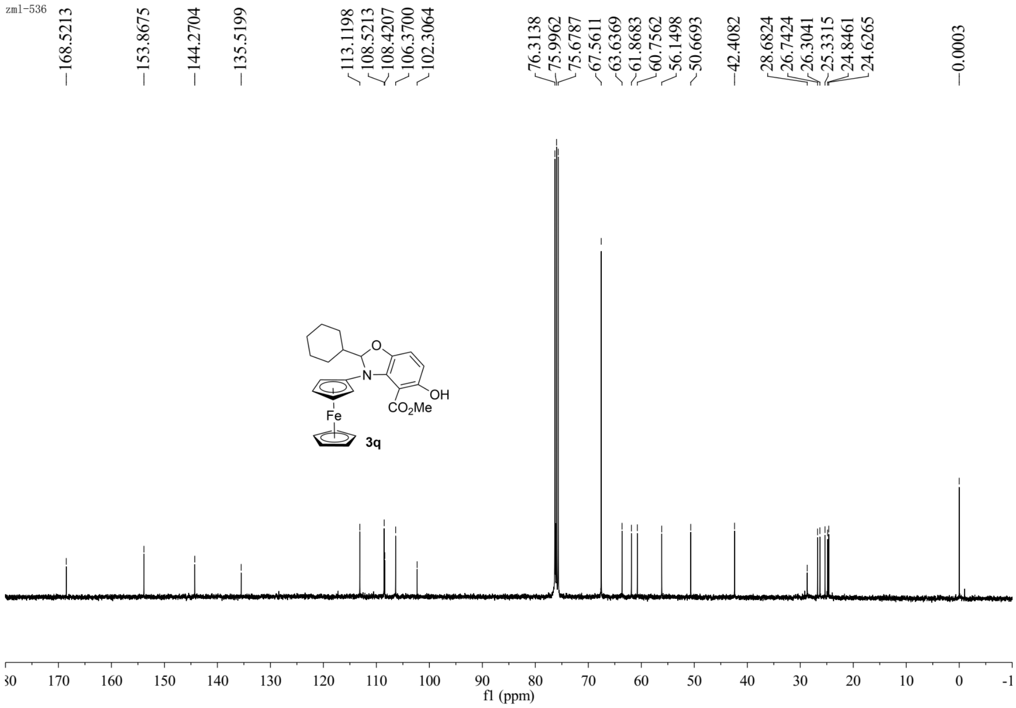

Supplement: Supplementary file 1 [file molecules-28-05615-s001.zip › molecules-2518864-supplementary.pdf]
